# Synapse fits neuron: joint reduction by model inversion

**DOI:** 10.1007/s00422-017-0722-1

**Published:** 2017-07-08

**Authors:** H. T. van der Scheer, A. Doelman

**Affiliations:** Mathematical Institute, Leiden University, P.O. Box 9512, 2300 RA Leiden, The Netherlands

**Keywords:** Conductance-based models, Synaptic transmission, Inverse systems, State-space realizations

## Abstract

In this paper, we introduce a novel simplification method for dealing with physical systems that can be thought to consist of two subsystems connected in series, such as a neuron and a synapse. The aim of our method is to help find a simple, yet convincing model of the full cascade-connected system, assuming that a satisfactory model of one of the subsystems, e.g., the neuron, is already given. Our method allows us to validate a candidate model of the full cascade against data at a finer scale. In our main example, we apply our method to part of the squid’s giant fiber system. We first postulate a simple, hypothetical model of cell-to-cell signaling based on the squid’s escape response. Then, given a FitzHugh-type neuron model, we *derive* the verifiable model of the squid giant synapse that this hypothesis implies. We show that the derived synapse model accurately reproduces synaptic recordings, hence lending support to the postulated, simple model of cell-to-cell signaling, which thus, in turn, can be used as a basic building block for network models.

## Introduction

### The need for (network) simplifications

In theoretical neuroscience, when modeling neurobiological systems, scientists are faced with a structural problem: “What details should be included, and what details should be left out?” Although nervous systems are studied at many levels of abstraction, there is no general agreement on which details matter, at a particular level, and which do not. Hence, with the increasing amount of neurophysiological knowledge gained, it can be tempting to include more and more detail in models of, e.g., nerve cell behavior. However, detailed models are not necessarily better (Dayan and Abbott 2001; Herz et al. 2006). For one, they need to be compared with data, and models with too many variables and parameters can often be made to fit almost any data. These may therefore be too general to provide any real insight. Second, the main reason for studying nerve cells is to better understand nervous systems, in particular, their functional role in adaptive, animal behavior. Yet, nervous systems typically consist of many thousands of neurons, while movements, such as head, eye, and limb movements, have relatively low degrees of freedom. In other words, starting from the neuron level, a working understanding or model of this functional role is still far a way. Not surprisingly, efforts to gain insight and to reduce the number of variables and parameters have resulted in elegant simplified neuron models (Knight 1972; FitzHugh 1961; Ermentrout and Kopell 1986; Hansel and Mato 2001; Izhikevich 2003; Touboul 2008). Still, even when composed of such simplified models, the analysis of networks (which from the neuron-level form the next level up) remains daunting, and hence, many simplifying assumptions are usually made in theoretical neuroscience (Dayan and Abbott 2001). These considerations lead us to the following questions. How can we simplify and reduce the number of variables and parameters without making too many assumptions? How to loose details without loosing the essentials? Where to start?

**Fig. 1:**
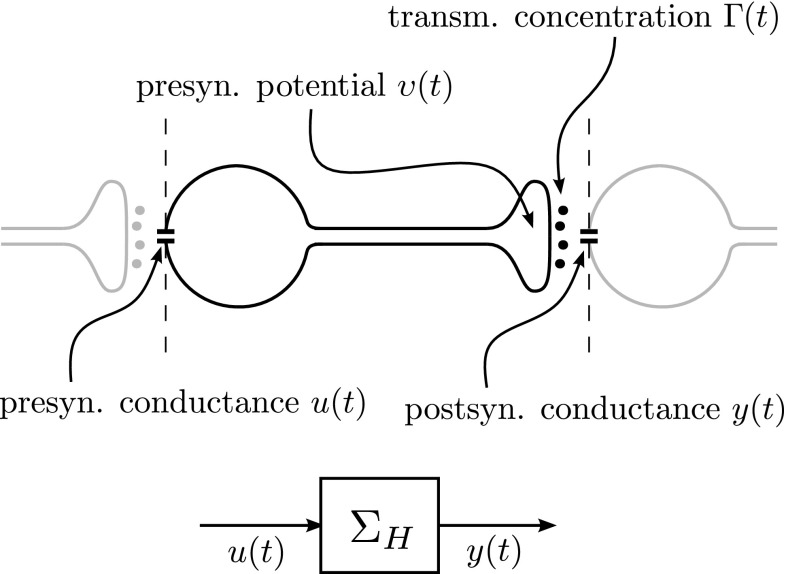
*Top* a schematic representation of a complete signal path from presynaptic conductance *u*(*t*) to postsynaptic conductance *y*(*t*). Two intermediate quantities are indicated along the way: the presynaptic potential $\upsilon (t)$, and the transmitter concentration $\varGamma (t)$. *Bottom* a ‘building block’ representation of the path from a functional input–output point of view

Since our goal is to reach a functional network level of abstraction that allows for network models with feedback connections, we will start by considering complete signal paths, that is, paths from conductance to conductance, from potential to potential, or from transmitter concentration to transmitter concentration (Fig. 1). This means that apart from neurons and their models (which typically only model an incomplete path from an input conductance or current to an output potential) we will have to consider synapses too. Once we have established valid models of such complete paths, we can use these as building blocks to ‘wire-up’ neurons into networks. We call a model of such a complete signal path a *high-level model* $\varSigma _H$. The question is: How can we obtain and validate such a model?

In this paper, we develop a novel procedure, partly inspired by Eliasmith and Anderson (2003), by which a candidate high-level model can be verified against data at the lower level. It involves an implicit matching constraint and applies to physical systems that can be viewed as converting some input *u* into some output *y* and that can be thought to consist of two subsystems connected in series such as the above complete signal path consisting of a neuron and a synapse. The aim of our method is to help find a simple, yet valid model of the full cascade-connected system, and the purpose of this high-level model is to describe the behavior of the full system from a functional point of view, stripped from details of implementation that, from an input–output perspective, could be considered inessential. Our method is specifically designed to help determine the appropriate level of detail by keeping the number of variables and parameters of the high-level model to a minimum.

### A novel approach to simplification

To start with, we introduce a few concepts and describe the situation to which our method applies. Consider a physical system as described above. Since it is natural to study complex systems through their subsystems, we make two reasonable assumptions.

**Fig. 2:**
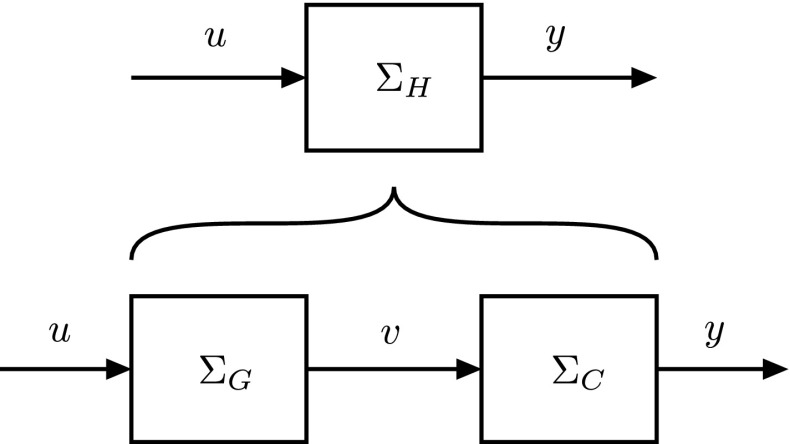
A schematic representation of a physical ‘input–output’ system that can be thought to consist of two subsystems connected in series. Our goal is to establish a simple, high-level model $\varSigma _H$, describing the behavior of the full system from a functional, input–output point of view. We assume that a model $\varSigma _G$ of the first subsystem is given, describing in more detail how part of the system is realized. What is thus still required, in order to complete the cascade, is a matching, complementary model $\varSigma _C$ of the second subsystem

**Fig. 3:**
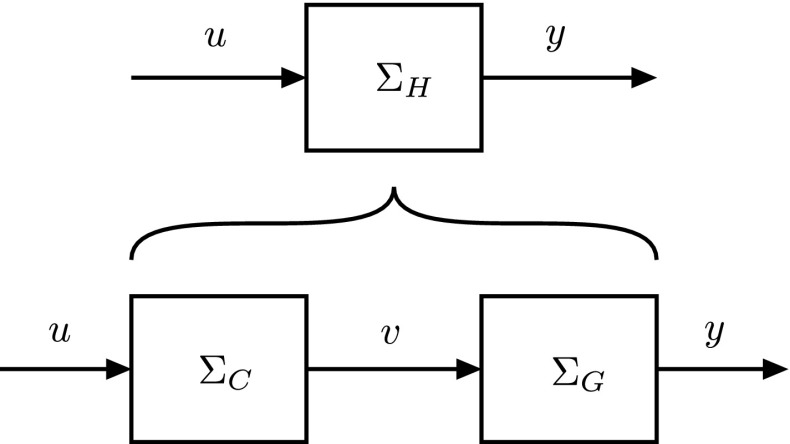
A schematic representation of a physical ‘input–output’ system that, as in Fig. 2, can be thought to consist of two subsystems connected in series. Again, our goal is to establish a simple, high-level model $\varSigma _H$, describing the behavior of the full system from a functional, input–output point of view, except now we assume that a model $\varSigma _G$ of the *second* subsystem is given, and we require a matching, complementary model $\varSigma _C$ of the *first* subsystem, in order to complete the cascade

1. We assume that a satisfactory model for one of the subsystems (e.g., the neuron) has already been established and is given, describing in more detail how part of the system is realized. We call this model of the identified subsystem the *given model* $\varSigma _G$.
2. We assume that measurement pairs $(u,\upsilon )$, relating the inputs and outputs of the first subsystem, and measurement pairs $(\upsilon ,y)$, relating the inputs and outputs of the second subsystem, are either available or relatively easy to obtain.

(Of course, if measurement pairs (*u*, *y*), relating the inputs and outputs of the full system, were available, we could try to model the desired higher level directly; however, for complex systems, this is generally not the case). What is thus still required, in order to complete the cascade, is a *matching* model of the remaining, unidentified subsystem, i.e., a model whose combination with the given model reduces to a simple, high-level model. We call such a matching model the *complementary model* $\varSigma _C$ (Figs. 2, 3). For instance, given a neuron model (the step from *u* to $\upsilon$ in Fig. 1) what is still required for a complete signal path is a complementary model of the synapse (the step from $\upsilon$ to *y* in Fig. 1). Or, vice versa, given a synapse model, what is still required is a complementary model of the neuron. Of course, in order to lead to a high-level reduction, the complementary model should not only match with the model already given; in addition, it should, by itself, also be *valid*. That is, it should agree with the data, so that its combination with the given model reduces to a high-level model that is valid *as well as* simple. How can this be achieved?

The solution to the above problem lies in adopting the scientific method: postulate a hypothesis and verify or falsify its validity. First of all, note that the complementary model could in principle be expressed abstractly in terms of the given model and the high-level model (as $\varSigma _C = \varSigma _H \circ \varSigma _G^{-1}$ in case of Fig. 2, or as $\varSigma _C = \varSigma _G^{-1} \circ \varSigma _H$ in case of Fig. 3). Of course, at this point, the simple, high-level model has not yet been established. In fact, this is exactly the model that we are after. The crucial idea now is, however, that this obstacle can be overcome by postulating a hypothesis. Our method can be summarized as follows: postulate a simple, high-level model $\varSigma _H$ of the full system and then use the inverse $\varSigma _G^{-1}$ of the given model to derive a verifiable, complementary model $\varSigma _C$ of the remaining, unidentified subsystem. In the case of a complete signal path, for instance, we first postulate a hypothetical model of cell-to-cell signaling and then use an appropriate, established neuron model to *derive* a complementary model of the synapse. The derived, complementary model can then be verified against measurements to either support or reject the hypothesis. As we shall show, the technical tools necessary to develop this idea, such as systems inversion, are already available in the literature.

We must stress that the complementary model introduced above is ‘merely’ a means to an end. In particular, it need not be simple itself. In fact, in most cases it will be quite the opposite and considerably abstract. Nevertheless, by construction, it is such that it can be verified against data. Furthermore, as our neurotransmitter example will show (Sect. 4.1), our method can also help with finding an equivalent physically meaningful realization for this derived, abstract model. More to the point however, the results from our squid example (Sect. 3.2) suggest that, at least in the case of cell-to-cell signaling, it is possible to obtain and validate simple, yet faithful, high-level models with our method, and these, after all, form our end goal.

Our method was developed with the neuron and synapse in mind, and, as outlined below, we will motivate and present our method with the aid of a relatively transparent, yet realistic example of a complete signal path that belongs to the squid’s giant fiber system. We also provide results for (small) networks with both excitatory and inhibitory connections, and our method may have even wider applicability. However, there are, of course, also other ways to reach the desired higher level, so in order to substantiate the claim that our method addresses a real need (which may not be immediately obvious) we now address some drawbacks associated with these alternative routes.

### The limitations of straightforward simplification

As another approach to reaching the desired, network level mentioned above, one could, in principle, complete the signal path by connecting an established neuron model in series with an established synapse model. Indeed, in the more or less traditional approach to neuronal modeling (Dayan and Abbott 2001) simplified neuron models (Izhikevich 2007) and simplified synapse models (Tsodyks and Markram 1997; Morrison et al. 2008; Rowat and Selverston 1993) are often developed independently from each other. This, however, introduces several problems. To begin with, in general, it does not result in the desired reduction, since the combined model will, almost always, be too elaborate for the desired network level of abstraction. Furthermore, an arbitrary combination of neuron model and synapse model is likely to be at least somewhat questionable, since it is well known that in cascades of nonlinear models (and synapses do also have interesting nonlinear features) even small parameter variations may lead to wildly different input–output behaviors. In other words, given the ever present small modeling ‘errors’ in both neuron model and synapse model (not to mention those introduced by independent simplification), it follows that this approach is vulnerable to misinterpretation. Furthermore, even if the combined model, in some way, allows for a simple, high-level interpretation, one could easily miss it. In fact, one could claim that choosing the ‘right’ combination of neuron model and synapse model requires prior knowledge, an interpretation of joint neuron-synapse behavior.

Alternatively, one could try to model the higher level, the joint input–output behavior of neuron and synapse, directly, more or less as in classical artificial neural networks, see, e.g., Hunt et al. (1992). In this case however, note that measurements may not be available for the complete signal path. In the case of the squid for instance, measurements are available for the membrane potential in response to an injected input current (Clay 1998) and for the postsynaptic current in response to presynaptic depolarizations of the membrane potential (Augustine et al. 1985), but not, at least not to our knowledge, for a complete path from conductance to conductance. In reality, setting up an experiment to isolate such a complete path may not even be feasible. In addition, in this direct approach, the question of how the joint behavior is realized physically would remain largely unanswered. Although, apart from these obvious alternatives, there are other ways to reach the higher network level, these generally require many assumptions, e.g., that neurons ‘encode’ their stimulus in a firing rate (Hopfield and Tank 1986; Hopfield 1984), that (within a localized subpopulation of neurons) the relevant, dynamic variable is the proportion of active cells per unit of time (Wilson and Cowan 1972), and so on (Chapeau-Blondeau and Chambet 1995; Abbott 1994; Dayan and Abbott 2001). Such assumptions are not always plausible.

### Our main (squid) example

In order to illustrate how our method can help reach the desired network level, we will use it to verify a hypothetical, high-level model of a complete signal path in a neuronal system that has been studied extensively, and for which an abundance of data is available, the squid giant fiber system. This system plays an important role in the jet-propelled escape responses of squid. For instance, a powerful single-jet escape response can be triggered by a sudden visual stimulus, a flash of light (Otis and Gilly 1990). Hence, a connection with observable, outward behavior has already been established and can serve as a starting point for our hypothesis.

**Fig. 4:**
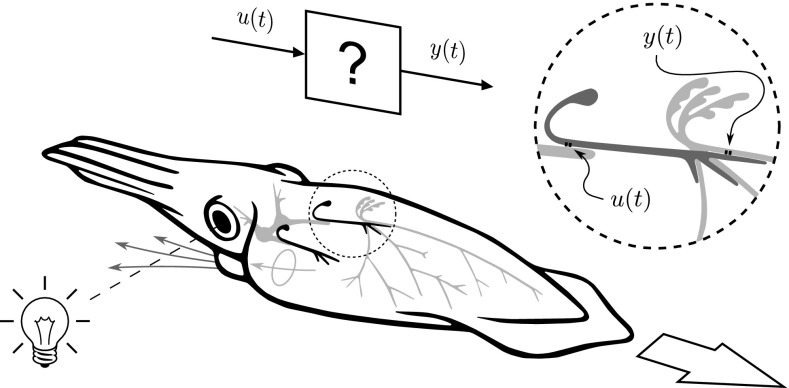
Simplified schematic representation of the squid giant fiber system in a single-jet, flash-evoked escape response: from the fused first-order giants (*gray*), to the paired second-order giants (*black*) via the stellate ganglion to the paired third-order giants (*gray*, only one side shown). One second-order giant is shown enlarged to indicate the complete path from presynaptic conductance *u*(*t*) to postsynaptic conductance *y*(*t*) that we seek to capture with a simple, yet valid, high-level model, in order to illustrate our method. (Based on various sources)

The giant cells that are involved in the single-jet, flash-evoked escape response of the squid are arranged in bilateral symmetry, and they include the giants that make up our complete signal path. Described briefly, the fused first-order giants receive their input from the optic lobe. These then synapse onto the paired second-order giants, which in turn (each on their respective sides) make synaptic contact with the axons of the third-order motor giants emanating from the stellate ganglion. Activation of the third-order giants causes the muscles in the mantle to contract and a jet of water to be expelled from the funnel (Otis and Gilly 1990). Our example of a complete signal path starts with the synaptic conductance input to the second-order giant and ends with the resulting postsynaptic conductance in the receiving third-order giant (Fig. 4).

**Fig. 5:**
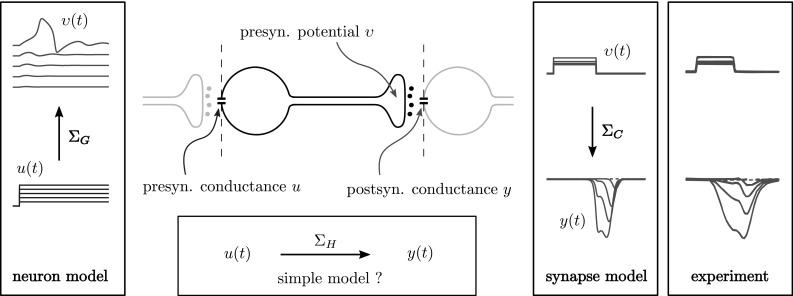
A pictographic summary of our method as applied to cell-to-cell signaling in the squid giant fiber system (cf. also Figs.  4, 7, and 8). Given a model $\varSigma _G$ of a squid neuron, it is possible to *derive* a complementary model $\varSigma _C$ of the squid giant synapse, by postulating a simple, high-level model $\varSigma _H$, describing what the giant fiber system does from a functional, input–output point of view. *Left* voltage responses $\upsilon (t)$ to step input conductances *u*(*t*) as produced by the FitzHugh-type neuron model $\varSigma _G$ given by (20). *Middle* the objective, a simple, high-level model $\varSigma _H$ of cell-to-cell signaling, represented here by a complete signal path mapping a presynaptic conductance input *u*(*t*) to a postsynaptic conductance output *y*(*t*). This is the system for which we *postulate* a hypothetical model (23). *Right* superimposed postsynaptic conductance responses *y*(*t*) to depolarizing presynaptic potentials $\upsilon (t)$ as produced by the *derived*, complementary synapse model $\varSigma _C$ given by (28), next to experimental data faithfully traced from Augustine et al. (1985). Positive conductances are plotted downwards

Given the central place that the above complete signal path takes in the squid’s giant fiber system, and given its implied role in escape behavior, it is tempting to interpret its input–output behavior as realizing an escape ‘threshold’. That is, as the total conductance input *u*(*t*) due to escape-initiating stimuli crosses a critical threshold, the output conductance *y*(*t*) suddenly becomes high and the squid ‘decides’ to escape. However, seemingly obvious interpretations may, of course, not be right. A model based on this view still needs to be validated or falsified. This is where our method comes in. We postulate a simple, high-level model based on an escape threshold (23), and then, given a FitzHugh-type model of a squid neuron (20), we use our method to derive the complementary synapse model (28) required to complete the signal path from *u*(*t*) to *y*(*t*) in Fig. 4 (remark 1). The dynamic responses of this derived synapse model (Figs. 5, 8) are in remarkable agreement with the measurements (Figs. 5, 7) reported in Augustine et al. (1985), hence lending support to the postulated simple model of cell-to-cell signaling. This indicates that simple, yet faithful, network-level models are feasible with our method.

A priori, it may seem unlikely that it is possible at all to reduce our complete signal path to a simple high-level model, given the intricate behaviors of the measured, intermediate quantities. What we must realize, however, is that these intricate behaviors may partly arise from requirements other than input–output function. They may be necessary to overcome noise, to conserve energy, to ensure that the signal travels from A to B, and so on. Hence, some of their features, although interesting in their own right, may not be essential from a functional input–output point of view. In other words, the ‘actual’ input-output transformation may be far more simple than the measurements initially suggest, as our results indeed demonstrate.

#### Remark 1

In the case of a complete signal path, we assume, in this paper, that a model of the neuron is given, since the conductance-based approach to neuron modeling has been widely accepted. One could also consider the case where a model of the synapse is given; however, the physiological effects of neurotransmitters and their mechanisms of release can vary considerably from transmitter to transmitter, and many mechanisms are questioned, debated or unknown (Langley and Grant 1997; Tauc 1997; Vautrin 1994; Vyskocil et al. 2009). For instance, in the case of the squid, many suitable neuron models are already available (Hodgkin and Huxley 1952; FitzHugh 1961; Rinzel 1985; Kepler et al. 1992; Clay 1998), while suitable models for the squid’s giant synapse seem less numerous (Llinás et al. 1976, 1981a, b). Note also that, in the case where the neuron model is given, our approach conveniently avoids unnecessary assumptions by circumventing the unknown mechanisms of neurotransmitter release just mentioned, yet, it does *not* ignore their dynamic effect.

### Physical realism: from basic cases to networks

A large part of the paper is concerned with establishing suitable classes of high-level models [Eqs. (19), (34), (39), (63), and (76)] from which hypotheses can be drawn. In particular, apart from the matching constraint mentioned earlier, we also impose a ‘physical’ realizability constraint on our models, so that our procedure results in complementary models and, therefore, in high-level models that are realistic. Hence, for hypothetical, high-level models drawn from these realizable classes (which are associated with classes of given models) the hard work of establishing a ‘physical’ realization of the complementary model has been done, and for these, our verification procedure can be followed without additional burden. In fact, for the squid’s complete signal path, we will explicitly follow our verification procedure, once we have established the realizable class (19). The more general of these classes are preceded by a motivational example and subsequently followed by another concrete example.

### Outline

The paper is structured as follows. First we set forth the general method in all its detail in Sect. 2. We distinguish between two cases, the case where a model of the first subsystem is given (Fig. 2), and the case where a model of the second subsystem is given (Fig. 3). We then introduce the ‘physical’ realizability constraint that our models need to satisfy in the interest of physical realism and illustrate the problems that this constraint introduces with a simple example on signal representation. In Sect. 3, we cover the case where a model of the first subsystem is given. We extend the example on signal representation to include more general signal transformations by complete signal paths and then apply the method to the squid giant fiber system, where we use it to derive the synapse model required to complete the signal path indicated in Fig. 4. (The more general case is treated in the appendix, along with a network example). In Sect.  4, we cover the case where a model of the second subsystem is given. We start with an example where a model of transmitter-dependent, postsynaptic conductance is given and then treat the more general multi-input multi-output case (with a network example, again, provided in the appendix). We end this section with some final remarks. Section 5 concludes the paper.

## A novel simplification procedure

In this section, we reiterate the main idea and go into it in more detail. We also introduce our additional realizability constraint. We then start our investigation with signal representation and give an illustrative example.

### The main idea: two cases to consider

As we stated before, the goal of our method is to help establish a simple, high-level model of a physical system that can be thought to consist of two subsystems connected in series and for which a model of one of the subsystems is already given (assumption 1). To this end, we provide a way to derive and validate a *complementary model* of the remaining, unidentified subsystem, where, as already mentioned, we distinguish between two cases. In the case where a model of the first subsystem is given (Fig. 2), our method can be described as follows:

1. We postulate a simple model $\varSigma _H$ for the full system at the desired, higher level, based on what we think the system does from a functional point of view. This model takes the form of an input–output dynamical system mapping time-varying inputs *u*(*t*) to time-varying outputs *y*(*t*).
2. We derive the complementary model in terms of the hypothesis and the given model as follows:
  - We derive a (left) inverse $\varSigma _G^{-1}$ of the given model. That is, we derive an inverse model which, when given the output $\upsilon (t)$ from $\varSigma _G$, reconstructs the input *u*(*t*) that caused it.
  - We connect the two systems $\varSigma _H$ and $\varSigma _G^{-1}$ in series to obtain the complementary model $\varSigma _C=\varSigma _H \circ \varSigma _G^{-1}$ of the *second* subsystem.
3. We verify the derived, complementary model $\varSigma _C$ (and, hence, indirectly the simple hypothesis) against measurements, which (by assumption 2) are available.

Of course, if the model is falsified, one can reiterate the process by updating the hypothetical, high-level model $\varSigma _H$, i.e., by returning to the first step. Similarly, in the case where a model of the second subsystem is given (Fig. 3), one derives a (right) inverse of the given model to obtain a verifiable complementary model $\varSigma _C=\varSigma _G^{-1} \circ \varSigma _H$ of the *first* subsystem.

As one iterates the above model-prediction loop, the key is to keep the model $\varSigma _H$ as simple as possible. This will not only facilitate further analysis, but will also help to avoid overfitting, since, if the hypothetical high-level model is too general, the complementary model can be *made* to fit virtually any data and such generality should be avoided. On the other hand, note that the complementary model is not only constrained by the data it is supposed to describe, but also by the high-level model and the data that the *given* model is supposed to describe, since, by its very construction, it incorporates the inverse of the given model. This, of course, also means that, if the high-level model is too simple, it will be impossible to find a reasonable fit (in which case a discrepancy between the data and the postulated hypothesis has been identified). In short, the high-level model should be general enough to approximate or mimic the data, but not so general as to fit almost any data.

Choices for the hypothetical, high-level model $\varSigma _H$ could be guided by theory (Kreinovich and Quintana 1991; Zhang and Sejnowski 1999), observations (Kouh and Poggio 2008), and even intuition. Classical examples of simplified, high-level representations in theoretical neuroscience include firing rate models or population models such as the Hopfield model (Hopfield 1984; Hunt et al. 1992) and the Wilson–Cowan model (Wilson and Cowan 1972). Such simplified representations can form the basis for network ‘modules’ in models at an even higher level of abstraction such as the modular models in Doya (1999); Doya et al. (2001) and Tin and Poon (2005).

### An additional constraint: realizability

Since we are interested in *physical* systems, our input–output models need to satisfy certain physical constraints, such as realizability and causality, in order to be realistic. Informally, an input–output system is said to be *realizable* if an input–output-equivalent state-space representation, called a *realization*, exists, cf. Kotta and Mullari (2006). Causality is an intrinsic property of input–output dynamical systems in state-space form (Khalil 2002), and not surprisingly, such state-space systems arise frequently as models of physical systems. Note however that, from an input–output perspective, the state variables of a system, or its details of implementation, are irrelevant. Any invertible state transformation results in a different realization of the *same* input–output mapping. Hence, for the hypothetical high-level model (Figs. 2 or 3), one could, in principle, consider direct mappings of the form $y=\varphi (u)$, where *u* is the input and *y* is the output, or input–output differential equations of the form ${\dot{y}} = \varphi (y,u)$, and so on. However, recall that we intend to use inverse models in our derivation, the input–output mappings associated with such inverse models are, in general, not realizable. Hence, in order to maintain physical realism, it is important to ensure realizability.

In line with our aim for physical realism, we will *demand* realizability of the derived, complementary model in this paper. This, in turn, will limit the choice of possible hypothetical models, as we shall see. In the case where a model of the first subsystem is given (Fig. 2), we impose that the complementary model has a state-space realization of the form: 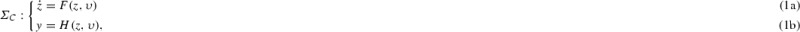


where *z*(*t*) is a suitably initialized, *k*-dimensional state, $F(\, . \, ,\upsilon )$ is a *k*-dimensional vector field parameterized by the time-varying input $\upsilon (t)$, and *H* is the output map, mapping the state and the input to the output *y*(*t*). Similarly, in the case where a model of the second subsystem is given (Fig. 3), we impose that the complementary model has a realization of the form: 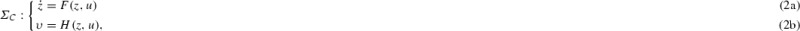


where now *u*(*t*) is the input and $\upsilon (t)$ is the output. Moreover, in the examples we provide in this paper, partly based on Abbott (1994) and FitzHugh (1961), realizations will be derived explicitly.

The constraint introduced above could, in principle, conflict with our aim for simplicity, since, in general, it is not satisfied by the inverse models that we intend to use in our derivation and this limits the choice of possible hypothetical models. Fortunately, however, it does not stand in the way of simple, high-level models with a simple interpretation, as the next example will show. Still, as a consequence of the above realizability constraint we cannot blindly follow the steps introduced in Sect. 2.1. Instead, for certain classes of given models, we will establish corresponding classes of high-level models from which hypothetical models can be drawn that *do* result in realizable complementary models, and for which the steps thus *can* be followed. For instance, we will follow the above steps explicitly, in our squid example, once we have established the class (19).

### An illustrative example: signal representation

As a first demonstration of our approach, and in order to show that the above realizability constraint limits the choice of possible hypothetical models, we start with an academic example. We consider the problem of recovering the input *u*(*t*) from the output response $\upsilon (t)$ of some given model $\varSigma _G$. This allows us to identify the problems we run into with one of the simplest possible equations. We focus on realizability. Instead of verifying a high-level hypothesis for some existing physical system, we consider whether or not it is possible, in *principle*, to complement the given model with another model $\varSigma _C$ in order to realize the most simple, high-level behavior $\varSigma _H$, the identity map:

3 $$\begin{aligned} y=u , \end{aligned}$$

with input *u*(*t*) and output *y*(*t*) (Fig. 2). Hence in this example, the verification step, which in a realistic setting would be part of our procedure, does not apply and is omitted. Nevertheless, as an added bonus, the example may already tell us something about graded non-impulsive neurons and about quiescent spiking neurons in their ‘subthreshold’ regime.

Consider the following problem. Let a model be given by:

4 $$\begin{aligned} \varSigma _G : \, {\dot{\upsilon }}= u + \beta (\upsilon ), \end{aligned}$$

where *u*(*t*) is the input, $\upsilon (t)$ is the state output for some initial value $\upsilon (0)$, and $\beta$ is some, possibly nonlinear, function. For instance, this model could represent the subthreshold part of an integrate-and-fire-type neuron model, where either $\beta (\upsilon )= \upsilon ^2$, in case of a quadratic integrate-and-fire model (Hansel and Mato 2001; Izhikevich 2007), or $\beta (\upsilon )= - \upsilon$, in case of a leaky, or forgetful, integrate-and-fire model (Knight 1972; Izhikevich 2007). (Note that integrate-and-fire-type models normally include a reset and that in the quadratic case, solutions can escape in finite time. Hence, in this case one would consider subthreshold inputs and initial values only, i.e., those inputs *u*(*t*) and initial values $\upsilon (0)$ that do not result in spiking, so that the reset of the model can be ignored). The problem we first want to address is as follows. Is it possible to recover the input *u* from the output $\upsilon$ of the given model, with a realizable complementary system $\varSigma _C$?

Note that in the above problem the required complementary model is the (left) inverse of the given model. That is, given the output $\upsilon (t)$ from $\varSigma _G$, we seek the system $\varSigma _C=\varSigma _G^{-1}$ that reconstructs the input *u*(*t*) that caused it. In this case, the cascade $\varSigma _C \circ \varSigma _G$ would reduce to the high-level identity map introduced above. By combining Eqs.  (3) and (4), we see that the inverse is given by:

5 $$\begin{aligned} \varSigma _C=\varSigma _G^{-1}: y= {\dot{\upsilon }} - \beta (\upsilon ) \; , \end{aligned}$$

where now $\upsilon (t)$ is the input and *y*(*t*) is the output.

We claim that the complementary model (5) does not have a classical state-space realization, i.e., it cannot be brought into the form (1). Hence, it does not satisfy our ‘physical’ constraint. In particular, note that in systems of the form (1) input derivatives, i.e., terms involving ${\dot{\upsilon }}$, do not appear. Given our aim for simplicity, and given the simplicity of the high-level identity map in this case, the proposed method would be useless if this problem could not somehow be resolved.

In order to overcome the above realizability problem, we alter the high-level identity map $y=u$ slightly and approximate it with the following system: 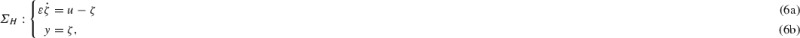


where $\varepsilon > 0$ and the state $\zeta (t)$ is initialized at a suitable point $\zeta (0)$. Note that this system is stable and that in the limit $\varepsilon \rightarrow 0$ (and for sufficiently well-behaved inputs *u*(*t*)) the identity is recovered. In this case, by combining (6) and (4), the corresponding complementary model $\varSigma _C=\varSigma _H \circ \varSigma _G^{-1}$ is given by: 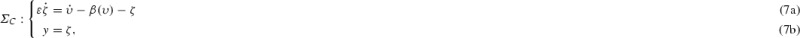


where now $\upsilon (t)$ is the input, *y*(*t*) is the output, and $\zeta (t)$ is the state.

Unlike (5), the complementary model (7) *can* be given a classical state-space realization. In fact by introducing the new variable $z=\psi _{\upsilon }(\zeta )=\varepsilon \zeta - \upsilon$ with $\zeta =\psi _{\upsilon }^{-1}(z) = (z + \upsilon ) / \varepsilon$ the system is explicitly brought into the classical state-space form (1): 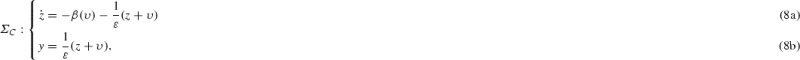


in which input derivatives ${\dot{\upsilon }}$ no longer appear. In other words, as a hypothesis for some physical system, this model would be more realistic than the previous exact inverse.

**Fig. 6:**
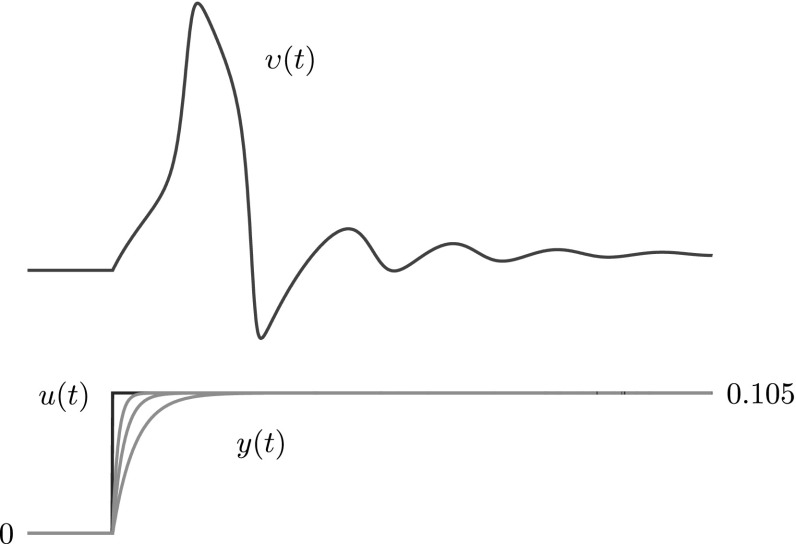
Several reconstructions *y*(*t*) of the step input conductance *u*(*t*) from the voltage response $\upsilon (t)$ of a neuron model. The realizable complementary model used for these reconstructions is based on the approximate ‘identity’ (6), which is of the form (12) with $h(\zeta )=f(\zeta )=\zeta$. Each reconstruction *y*(*t*) corresponds to a different value of the parameter $\varepsilon$ in this approximate identity. Note that we can get arbitrarily close to the original input provided that we choose the parameter $\varepsilon$ sufficiently small

In sum, although in the quadratic integrate-and-fire case, for instance, both $\varSigma _G$ and $\varSigma _C$ are nonlinear, their cascade reduces to a simple, in this case even linear $\varSigma _H$. Furthermore, by taking $\varepsilon >0$ small enough, the above complementary system can recover sufficiently well-behaved inputs *u*(*t*) with any desired degree of accuracy. Since the given model above includes the subthreshold part of integrate-and-fire-type models, and since we will later extend this result to a general class of single-compartment models (Sect. 3, Fig. 6), this arbitrary degree of accuracy may point to the possibility that in ‘stubby’ neurons with short axons even ‘subthreshold’ stimuli are well represented. If so, then, in local circuits of neurons with short processes, subthreshold signal processing may play a larger role than is sometimes assumed.

The above is an example of the case where a model of the first subsystem is given and there are two things to note: (1) not every high-level model results in a realizable complementary model, and (2) if a realization does exist, then finding it involves finding a change of variable $z=\psi _{\upsilon }(\zeta )$ that depends on the input $\upsilon$. The latter is inherent to the case where a model of the first subsystem is given and such a change of variable is an example of a *generalized* state transformation or *extended* state coordinate transformation (Kotta and Mullari 2005). In general, finding a transformation that results in a realization involves solving a system of PDE’s (Glad 1989). Fortunately, however, for numerical verifications of hypotheses, establishing the existence of a realization is sufficient, since in off-line verification the future iterates that appear in the numerical inverse are available (e.g., the inverse of $\upsilon _{t+1}=f_{\upsilon _t}(u_t)$ becomes $u_t = f_{\upsilon _t}^{-1}(\upsilon _{t+1})$, if it exists).

The case where the second subsystem is given, in a sense, is easier, as we shall see in Sect. 4. Although finding an explicit realization may (or may not) involve solving an implicit function, it does not require a generalized state transformation. Furthermore, the results are more general. We start however, with the case where a model of the first subsystem is given.

## The first subsystem is given

In this section, we cover the case where a model of the first subsystem is given. We first extend the results obtained for signal representation above to a more useful class of single-compartment, conductance-based neuron models, and at the same time we take our first step toward signal transformation. This forms the bases for our next, and main example, the squid giant fiber system.

### A representative example: conductance-based models

Consider the following problem. Let a neuron model be given by a single-compartment, conductance-based model of the fairly general form: 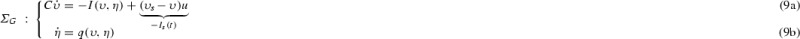


where the output $\upsilon (t)$ represents the membrane potential, the parameter *C* represents the membrane capacitance, and the input *u*(*t*) represents the total synaptic conductance, resulting in a synaptic current $I_s(t)$ with driving force $\upsilon _s-\upsilon$ and reversal potential $\upsilon _s$. (We will deal with distinct ion-specific reversal potentials and conductances in Sects. A.2, A.3). The other membrane current $I(\upsilon , \eta )$ represents the total of all remaining voltage-dependent ionic currents, and the other state variables $\eta =(\eta _1, \ldots , \eta _n)$ represent activation and inactivation variables, often called gating variables. We assume that the model has a stable rest state for $u(t) \equiv 0$ and that it is initialized at rest. The problem we now want to address is as follows. What class of hypothetical high-level models $\varSigma _H$ from conductance to conductance (Fig. 1) would result in a realizable complementary synapse model $\varSigma _C$?

In order to tackle the above problem, we first derive the inverse of the given model. For $\upsilon \ne \upsilon _s$, the model has an inverse given by: 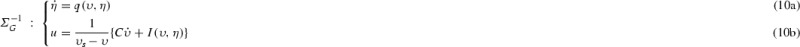


where now $\upsilon (t)$ is the input, *u*(*t*) is the output, and $\eta (t)$ is the suitably initialized state. Note the input derivative ${\dot{\upsilon }}$ in the output Eq. (10b). We claim that this inverse system does not have a classical state-space realization. Note also that the output *u*(*t*) of this inverse system depends on the solution $\eta (t)$ of the forced system (10a), driven by $\upsilon (t)$. In particular, it depends on the initial conditions $\eta (0)$. Since the internal state $\eta (t)$ of the given model $\varSigma _G$ will not be available from the output $\upsilon (t)$ of $\varSigma _G$, we assume that the inverse system is sufficiently stable, so that solutions with different initial conditions all converge to the same solution. This may seem restrictive, but, as shown, e.g., in Röbenack and Goel (2007), it is typically satisfied by the activation and inactivation dynamics:

11 $$\begin{aligned} {\dot{\eta }}=[\tau (\upsilon )]^{-1} \{\eta _{\infty }(\upsilon )-\eta \}=:q(\upsilon ,\eta ) \end{aligned}$$

of neuron models, where $\tau (\upsilon )$ is a diagonal matrix of positive, voltage-dependent time constants, and where each component of the steady-state solution $\eta _{\infty }(\upsilon )$ is a monotonic function of $\upsilon$.

We now extend the previous result on signal representation to include high-level models other than the approximate identity map (6). Our aim is to establish a larger class of hypothetical, high-level models, for which a realization of the complementary model $\varSigma _C=\varSigma _H \circ \varSigma _G^{-1}$ can be derived explicitly. Such complementary models can then be used as synapse models, so that hypotheses for cell-to-cell signaling, drawn from the proposed class of high-level models, can be tested against synaptic data, which is exactly what we will do in our squid example. We first consider the subclass consisting of high-level models of the form: 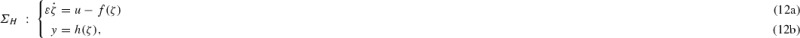


where $\zeta (t)$ is a suitably initialized, one-dimensional state, *u*(*t*) is an input conductance, *y*(*t*) is an output conductance (Fig. 1), and *f* and *h* are appropriate maps. Note that this class includes the previous approximate identity (6) with $h(\zeta )=f(\zeta )=\zeta$. Note also that the suggestive parameter $\varepsilon >0$ is only relative. In particular, it can be scaled away by a rescaling of time.

Combining Eqs. (10) and (12), we find that the complementary synapse model $\varSigma _C=\varSigma _H \circ \varSigma _G^{-1}$ is given by: 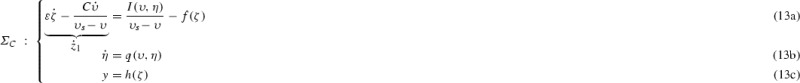


where the presynaptic potential $\upsilon (t)$ is the input, the postsynaptic conductance *y*(*t*) is the output, and $(\zeta (t),\eta (t))$ is the state. Note that this system is not yet of the form (1); it still contains input derivatives ${\dot{\upsilon }}$. As before however, and as already indicated by the introduction of the ${\dot{z}}_1$-label above, this problem can be alleviated by a coordinate change.

In order to convince ourselves that the complementary synapse model $\varSigma _C$ could, in principle, be realized by a physical system, we seek an equivalent state-space realization of the form (1) in *z*-coordinates in which input derivatives no longer appear. To this end, we introduce a coordinate transformation $\varPsi _{\upsilon }$ of the form:14that changes only a single coordinate, the first coordinate. In other words, except for a new name or label, the other $\eta$-coordinates remain unchanged. The transformation is further specified by:

15 $$\begin{aligned} z_1=\psi _{\upsilon }(\zeta ) = \varepsilon \zeta + C\ln |\upsilon _s-\upsilon | , \end{aligned}$$

and its inverse $\varPsi ^{-1}_{\upsilon }(z)$ is determined by:

16 $$\begin{aligned} \zeta =\psi _{\upsilon }^{-1}(z_1)=\frac{1}{\varepsilon }\{z_1-C\ln |\upsilon _s-\upsilon |\} . \end{aligned}$$

One can check that, for $\upsilon \ne \upsilon _s$ (remark 2), this transformation results in an explicit realization of the complementary synapse model, given by: 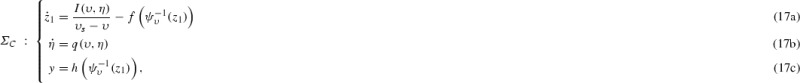


or 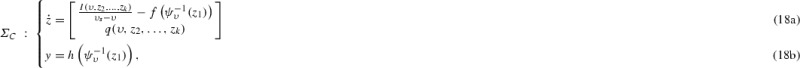


which is clearly of the form (1) and, hence, no longer contains input derivatives. In order to underline the value of this result, note that this state-space model is indeed such that the nonlinear cascade $\varSigma _C \circ \varSigma _G$ reduces to $\varSigma _H$. Furthermore, one is still ‘free’ to postulate any hypothetical high-level model from the subclass (12) by specifying *f*, *h*, and $\varepsilon$. When verified, such high-level models can be used in networks with feedback connections.

The above immediately extends the result obtained in our illustrative example (Sect. 2.3) to the general class of neuron models (9). That is, given the output response of *any* neuron model from this general class, we can reconstruct the input conductance that caused it with any desired degree of accuracy, by using the complementary model that corresponds to the approximate identity (6), see Fig. 6 (remark 3). (Here we used the neuron model from our main squid example in Sect.  3.2 for convenience). Again, since the class (9) represents spiking and graded non-impulsive neurons alike, this arbitrary degree of accuracy may point to the possibility that in ‘stubby’ neurons even ‘subthreshold’ stimuli are well represented. Of course, the reason for using the approximate identity here and in our previous example is not its plausibility or realism; we will get to that in our main example, it is to show that (should the data support it) our method allows for one of the simplest possible reductions. Our aim, after all, is simplicity. In addition, note that the full cascade behaves as one would expect from the simple ‘hypothesis’ (6) and, as a consequence, how severely this simplicity constrains the dynamic behavior of the complementary model in response to the modeled nerve impulse. Clearly, in ‘shaping’ the response, the single parameter $\varepsilon$ provides only one degree of freedom. This illustrates why keeping the number of parameters in the high-level model to a minimum will tend to avoid overfitting.

It is straight forward to generalize the above results for the subclass of models of the form (12) to an even more general class of high-level models of the form: 
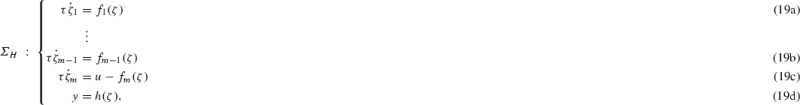


where $\zeta (t)$ is now an *m*-dimensional state and the output *y*(*t*) consists of an arbitrary number of distinct output conductances. Recall however that the key is to keep the hypothetical high-level model as simple as possible. (Again, the parameter $\tau$ is only relative and can be removed by a rescaling of time). We use this idea to verify a hypothetical high-level model for the complete signal path in the squid next.

#### Remark 2

When the invertibility condition $\upsilon \ne \upsilon _s$ is violated, some input information may be lost and we have to give up the possibility of perfect recovery or lossless representation in the case of a single conductance input. On the other hand, when multiple, distinct conductances are taken into account (Sects. A.2, A.3), the corresponding invertibility conditions are unlikely to ever be violated. Furthermore, even in the single-input case above, there is still a way for nature to avoid a violation of the invertibility condition: the synaptic reversal potential $\upsilon _s$ could lie outside the normal range of membrane potentials. Moreover, even when the condition is violated (e.g., during the upstroke and downstroke of an action potential), then the loss of information could still be minimal. Indeed, the rate of change $| {\dot{\upsilon }} |$ near $\upsilon =\upsilon _s$ determined by $\left| I(\upsilon _s, \eta ) \right|$ in (9) could be large or maximal, so that the membrane potential $\upsilon$ spends a minimal amount of time there.

#### Remark 3

Although in this paper we derive explicit realizations for our examples, for numerical verifications of hypotheses such explicit realizations are not required. It is sufficient to establish their existence. Hence, in the input-reconstruction example, and our main squid example, we used the more generally applicable numerical analogue provided in the appendix, Sect. A.1.

### Main example: the squid giant fiber system

In order to illustrate our approach, we now apply the realizability results above and use our method to verify a hypothetical, high-level model of a complete signal path in the squid giant fiber system. The aim of our example is to convince the reader that the method is useful and to show that simple, yet faithful high-level models are feasible. Our complete path starts with the synaptic conductance input to the second-order giant and ends with the resulting postsynaptic conductance in the receiving third-order giant (Fig. 4).

Since we use existing data (instead of data tailored to our need), we replace one of our previous assumptions with a slightly stronger assumption. Recall that, in order to apply our method, we assume: (1) that a satisfactory model for one of the subsystems, the neuron, is already given and (2) that measurements for the subsystems are available in the form of input–output pairs. Unfortunately, the second assumption, to our knowledge, is not completely satisfied. Although it is likely that L-glutamate is a transmitter at the squid giant synapse (Messenger 1996), there may be others, and this forces us to lump all transmitter-dependent conductances together in one total synaptic conductance, with one reversal potential. The assumption that this is a valid simplification replaces the second assumption above, since it allows us to use existing data. Although such a stronger assumption should, in general, be avoided, the fact that we *can* use existing data is a strong plus. It emphasizes our aim to summarize data with theory.

We can sum up the problem we want to address as follows.

- Our aim is to establish a simple, high-level model $\varSigma _H$ from input conductance *u*(*t*) to output conductance *y*(*t*) describing what the complete signal path does from a functional point of view (Fig.  4 and remark 4).
- We can take a FitzHugh-type neuron model $\varSigma _G$ as given, describing the voltage response $\upsilon (t)$ of the second-order giant to an input conductance *u*(*t*). Here we assume that we can lump together all synaptic input conductances converging onto the second-order giant into one total synaptic input conductance *u*(*t*) with one synaptic reversal potential $\upsilon _s$.
- What we seek, in order to complete the signal path (Fig. 2), is a complementary model $\varSigma _C$ of the squid giant synapse, that is a model from presynaptic potential $\upsilon (t)$ to postsynaptic conductance *y*(*t*).

Our method allows us to derive this complementary synapse model as follows (Fig. 5):

1. We postulate a hypothetical, high-level model $\varSigma _H$, drawn from the class (19), and based on what we know about squid.
2. We derive the complementary model in terms of this hypothesis and the inverse $\varSigma _G^{-1}$ of the given neuron model.
3. We verify the derived synapse model $\varSigma _C=\varSigma _H \circ \varSigma _G^{-1}$ against the measurements reported in Augustine et al. (1985).

It is important to note that, since the model of the squid giant synapse $\varSigma _C$ incorporates the inverse of the given neuron model, it is not only constrained by the measurements in Augustine et al. (1985), but also by the typical nerve impulses observed in squid giant axons (Clay 1998).

*The given neuron model* There are many candidate neuron models to choose from. The model $\varSigma _G$ that we use to describe in more detail how part of the high-level model is realized is of the form (9) and is based on the FitzHugh model (FitzHugh 1961; Izhikevich 2007). It is given by:

20a $$\begin{aligned} C {\dot{\upsilon }}&= \overbrace{\kappa (\upsilon \! - \! \upsilon _r)(\upsilon \! - \! \upsilon _t)(\upsilon \! - \! \upsilon _p) - \eta }^{-I(\upsilon ,\eta )} + \overbrace{(\upsilon _s \! - \upsilon )u}^{-I_{s}(t)} \end{aligned}$$

20b $$\begin{aligned} {\dot{\eta }}&= \frac{1}{\tau _{\eta }} \{ \lambda (\upsilon \! - \! \upsilon _r)-\eta \} =: q(\upsilon ,\eta ) \end{aligned}$$

where $\upsilon (t)$ is taken to represent the membrane potential and $\eta (t)$ is a recovery current. The parameter *C* represents the membrane capacitance, the parameter $\upsilon _r$ is the resting potential, the parameter $\upsilon _t$ is the instantaneous threshold potential, cf. Izhikevich (2007), and $\upsilon _p$ roughly corresponds to the peak potential. The input that usually characterizes a *current* in the FitzHugh model has been replaced by a synaptic current $I_{s}(t)$ with driving force $\upsilon _s-\upsilon$ and synaptic reversal potential $\upsilon _s$; hence, the input *u*(*t*) is now a *conductance* input. The parameter $\tau _{\eta }>0$ is a time constant, and the other parameters $\kappa$ and $\lambda$ are assumed to have appropriate dimensions. Note that strictly speaking the FitzHugh model is a model of the squid giant axon, a third-order giant, not a second-order giant.

*Step 1: A high-level model based on escape* The powerful single-jet escape response of squid can be triggered by a sudden visual stimulus, a flash of light (Otis and Gilly 1990). It is an example of what is often called a fixed action pattern, that is, as a releasing stimulus crosses a certain critical threshold a stereotyped, behavioral pattern is elicited with full strength. (Non-cephalopod examples of such fixed acts include sneezing and vomiting). The giant neurons involved in such escape responses are often viewed as decision-making interneurons: preprocessed sensory information converges onto the cell and the giant fiber responds in a threshold-type fashion (Reichert 1992; Dorsett 1980).

The first hypothetical model $\varSigma _H$ that we found to agree with the measurements is based on viewing the inflection point of a bounded *S*-shaped function:

21 $$\begin{aligned} y \approx S (\mu (u - \rho ) ) \; , \end{aligned}$$

as an escape threshold. Here $\mu >0$, and *S* is the standard logistic function:

22 $$\begin{aligned} S(x)=\frac{1}{1 + e^{-x}} \; . \end{aligned}$$

This initial threshold hypothesis is influenced by the units in artificial neural networks. By construction, we take it that, as the total, presynaptic conductance due to preprocessed, escape-initiating stimuli crosses the critical threshold $u(t)>\rho$, the postsynaptic conductance *y*(*t*) suddenly becomes high, and the squid ‘decides’ to escape. Although this view (which is almost implicit in biological texts) is very simple, the model based on this view, below, agrees remarkably well with the available experimental data.

Unfortunately, the crude threshold function (21), which forms the basis for our hypothesis, is not yet of the form (19). Hence, in order to ensure realizability of the complementary synapse model $\varSigma _C$, we repeat the trick in the introductory example, three consecutive times this time (remark 5), and approximate the initial hypothesis with: 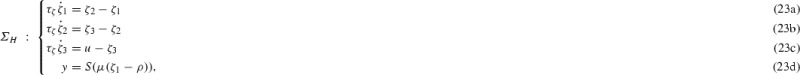


where $\tau _{\zeta }>0$, and in the limit $\tau _{\zeta } \rightarrow 0$, the initial hypothesis (21) becomes exact. The system (23) *is* of the form (19), and it follows that a state-space realization (1) of the resulting, complementary model $\varSigma _C$ can be derived explicitly. In fact, we will derive a realization of this synapse model shortly.

If verified, the hypothetical model (23) represents a significant simplification over composite models based on physiology alone. It has only three parameters, a parameter $\rho$ for shifting the threshold of the output map, a parameter $\mu > 0$ for adjusting its slope, and a parameter $\tau _{\zeta }$ for adjusting the amount of input smoothing. (The latter can be scaled away by a rescaling of time). Note that it is a Wiener model, i.e., a linear dynamical system followed by a nonlinear static map (Hunter and Korenberg 1986; Henson and Seborg 1997). Such models are frequently used as ‘processing units’ or ‘nodes’ in artificial neural networks (Hunt et al. 1992) and are members of a larger class of cascade models (Hunter and Korenberg 1986; Herz et al. 2006). Their appeal lies in their conceptual simplicity; the resulting abstract networks are amenable to analysis and hence facilitate the derivation of learning rules. Of course, we do not expect such a simple hypothesis to result in exact agreement with the measurements. Our aim is to show that the *method* is useful.

*Step 2: The complementary synapse model* In order to derive the resulting complementary synapse model, note that, since our given model (20) is of the general form (9), its stable inverse is given by (10). Hence, combining Eqs. (10) and (23), we find that the complementary synapse model $\varSigma _C=\varSigma _H \circ \varSigma _G^{-1}$ is given by: 
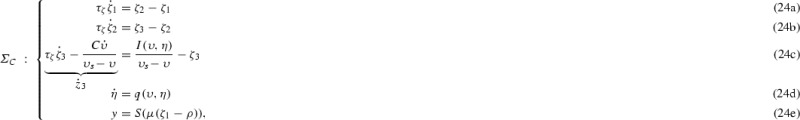


where the presynaptic potential $\upsilon (t)$ is the input, the postsynaptic conductance *y*(*t*) is the output, and $(\zeta (t),\eta (t))$ is the state. As before, and as already indicated by the ${\dot{z}}_3$-label, this model can be given a state-space realization by changing coordinates.

*An explicit realization of the synapse model* In order to convince ourselves that the complementary synapse model $\varSigma _C$ could, in principle, be realized by a physical system, we, once again, seek a state-space realization of the form (1) in *z*-coordinates. As before, we introduce a coordinate transformation $\varPsi _{\upsilon }$ of the form:25that changes only a single coordinate, the third coordinate, i.e., the other coordinates remain unchanged. The transformation is further specified by:

26 $$\begin{aligned} z_3=\psi _{\upsilon }(\zeta _3)=C\ln |\upsilon _s-\upsilon | + \tau _{\zeta }\zeta _3 \; , \end{aligned}$$

and its inverse $\varPsi _{\upsilon }^{-1}(z)$ is determined by:

27 $$\begin{aligned} \zeta _3=\psi _{\upsilon }^{-1}(z_3)=\frac{1}{\tau _{\zeta }} \Big \{z_3-C\ln |\upsilon _s-\upsilon |\Big \} \; . \end{aligned}$$

For $\upsilon \ne \upsilon _s$, this transformation results in an explicit realization of the complementary synapse model, given by: 
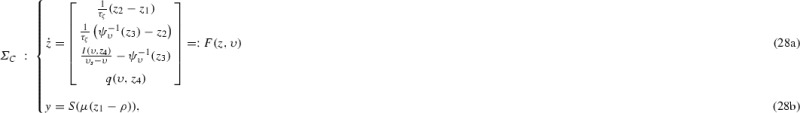


which is clearly of the form (1).

In order to underline the value of our result, note that the state-space realization derived above is such that the nonlinear cascade $\varSigma _C \circ \varSigma _G$, given by (20) and (28), reduces to $\varSigma _H$, given by (23). This high-level model has a very simple interpretation and, if validated, it leaves out a lot of the biophysical detail, without loosing the input–output essentials. Of course, the realization of the synapse model (28) is highly abstract and one may want to relate its variables to quantities other than the presynaptic potential and the postsynaptic conductance, such as the neurotransmitter concentration. Our method indeed also allows for this, and we will show how in Sect. 4.1. However, since we are unaware of any documented transmitter time courses recorded at the squid giant synapse, we will not include the transmitter concentration in our validation of the model.

**Fig. 7:**
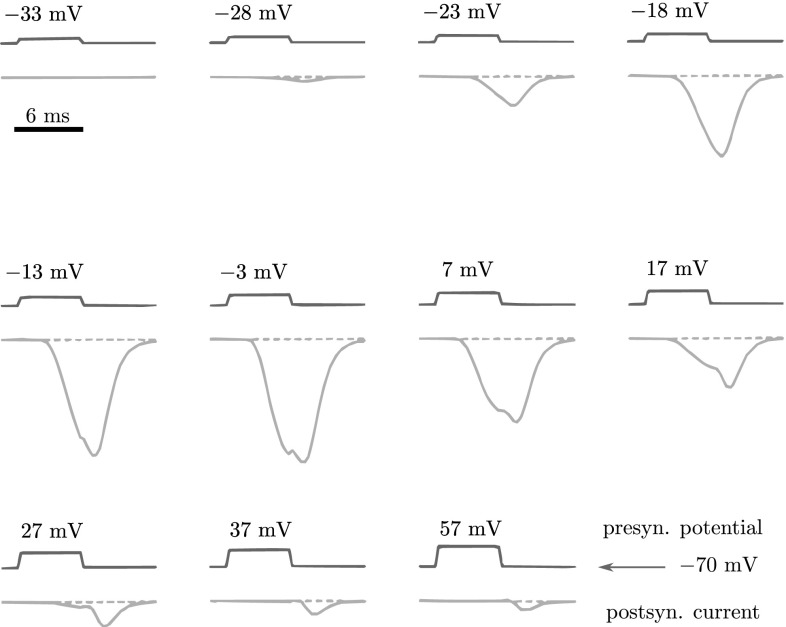
Recordings from the squid giant synapse: post synaptic currents (*lower traces*) in response to presynaptic depolarizing, 6 ms pulses (*upper traces*) from a holding potential of ${-}$70 mV. Faithfully traced from Augustine et al. (1985)

**Fig. 8:**
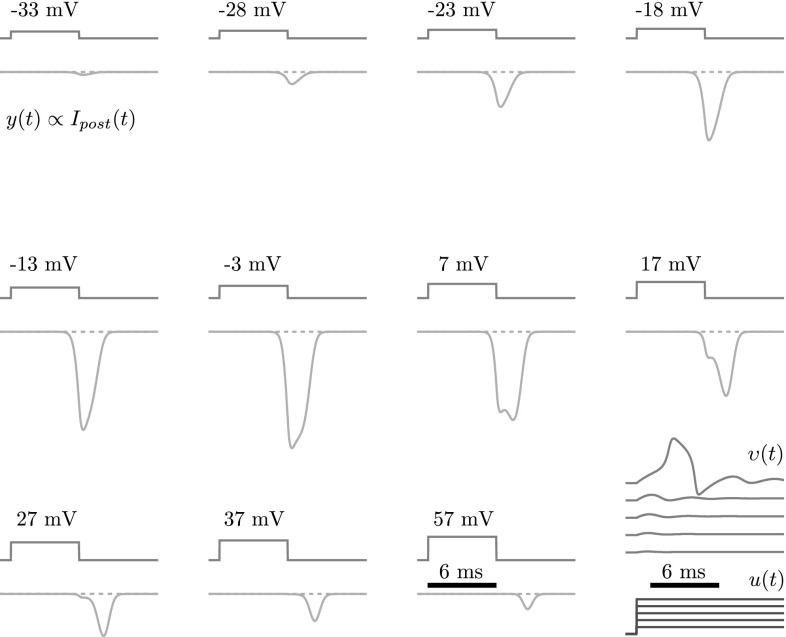
An approximate reproduction of the measurements in Augustine et al. (1985) by our theoretically derived model $$\varSigma _C : \upsilon \rightarrow y$$, where the postsynaptic conductance *y*(*t*) is taken to be proportional to the postsynaptic current $$I_{post}(t)$$ in the original figure, cf. Eq. (29). Presynaptic depolarizing pulses $$\upsilon (t)$$ are as in the original figure except that the parameters below are for equivalent decivolts. Also shown in the lower right corner are voltage responses $$\upsilon (t)$$ of the neuron model $$\varSigma _G$$ to step input conductances *u*(*t*), in 0.021 increments from zero, for the same parameters and timescale. The voltage responses for the more traditional step input *currents* (not shown) are very similar. Parameters of the FitzHugh-type neuron model $$\varSigma _G$$ and its inverse: $$C = 1 $$, $$\kappa = -1.38$$, $$\upsilon _r = -0.69$$, $$\upsilon _t = -0.52$$, $$\upsilon _p = 2.42$$, $$\upsilon _s = 4.7$$, $$\tau _{\eta } = 1$$, $$\lambda = 3.44$$. Parameters of the hypothesis $$ \varSigma _H $$: $$\tau _{\zeta }=\tau _{\eta }$$, $$\mu =130$$, $$\rho =0.201$$. Initial conditions: $$\zeta _1(0)=\zeta _2(0)=\zeta _3(0)=\eta (0)=u(0)=0$$ and $$\xi (0)=\upsilon _r$$. (Note that the timescale of processing $$\tau _{\zeta }=\tau _{\eta }$$ at the desired network level agrees with that of the ‘slow’ recovery dynamics (20b) of the neuron, not that of the ‘fast’ membrane dynamics)

*Step 3: Verification* In order to verify the complementary model $\varSigma _C$ (and hence, indirectly, the hypothetical high-level model), we compare it to the measurements reported in Augustine et al. (1985), which are faithfully redrawn in Fig. 7 for our convenience. During the original experiment, postsynaptic currents were recorded, while the presynaptic potential was depolarized by brief 3–6 ms pulses. The postsynaptic potential was held constant near its rest potential. The pulses were separated by at least 60 s, and their duration was deliberately kept short to avoid adaptation such as synaptic depression or facilitation.

As with the actual synapse in the experiments, the model $\varSigma _C$ is subjected to a series of presynaptic depolarizing pulses $\upsilon (t)$. The model $\varSigma _C$ then converts these into postsynaptic conductances *y*(*t*) (Fig.  8). In order to compare these with the postsynaptic *currents* in Fig. 7, we recall that, in the original experiment, the postsynaptic potential of the giant axon was held constant at its resting potential $\upsilon _r \,$. Hence, since we lump all transmitter-dependent conductances together in one total postsynaptic conductance with one synaptic reversal potential $\upsilon _s \,$, we can take this total conductance *y*(*t*) to be proportional to the postsynaptic current:

29 $$\begin{aligned} I_{\mathrm{post}}(t)=y(t)\underbrace{\{\upsilon _r - \upsilon _s \}}_{\mathrm{constant}} \; \propto \quad y(t). \end{aligned}$$

In other words, the output conductances *y*(*t*) of the model shown in Fig. 8 can be compared directly with the recorded postsynaptic currents $I_{\mathrm{post}}(t)$ shown in Fig. 7.

Due to the simplicity of the hypothesis, it is not easy to find parameters that agree with the measurements (remark 6). In fact, one of the main goals of our method is to ensure that the interdependent models are sufficiently constrained by the data. Since the synapse model $\varSigma _C$ shares the parameters of the neuron model $\varSigma _G$, these must not only be chosen such that $\varSigma _C$ agrees with the measurements in Augustine et al. (1985), but also such that the neuron model $\varSigma _G$ generates a nerve impulse for certain step input conductances (and more traditionally step input currents). Hence, these parameters are constrained by both synapse behavior (Augustine et al. 1985) *and* squid axon behavior (Clay 1998).

Despite the heavy constraints just mentioned, the results come strikingly close (compare Figs. 7, 8) especially given the fact that the synapse model $\varSigma _C$ was *derived* from a simple hypothesis! Note the characteristic features in the results. As in the original experiment, the postsynaptic conductance *y*(*t*), produced by the model, first grows steadily around the ‘off’ command with each increasing, depolarizing, presynaptic pulse $\upsilon (t)$. Then, a dimple starts to appear around the ‘off’ commands, resulting in a bimodal conductance response. As the pulses are increased further, the pre-dimple part of *y* diminishes until it dies out completely. The post-dimple part also diminishes, but slower, until, eventually, only a small after-effect remains. Note that we did not explicitly account for these features in our simple hypothesis. They arise from, and can be completely attributed to, our use of the inverse neuron model. Hence, these results are indicative of the potential predictive power of our approach.

In order to contrast our method with the more traditional approach (Sect. 1.3), consider, now, one of the simplest independently obtained synapse models:

30 $$\begin{aligned} \tau {\dot{y}} = -y + w S \left( \mu (\upsilon -\rho ) \right) , \end{aligned}$$

where *S* is as in (22), and the parameters $\tau$, *w*, $\mu$, and $\rho$ have appropriate dimensions (Rowat and Selverston 1993). Despite its simplicity, this model, combined with the neuron model, does not result in a simpler high-level model, and, even though it is based on data obtained from the squid giant synapse (Katz and Miledi 1967), it also does not explain the dimple, i.e., the bimodal synaptic response (Fig. 7). In fact, explicit modeling of this feature with independent models would only move us further away from a high-level simplification. This underlines one of our guiding principles, i.e., that joint simplification under interdependent constraints is preferable over independent simplification. In our approach, neuron and synapse are ‘in tune’ with one another, expressed by their shared model parameters, in order to carry out their joint functional behavior, expressed by $\varSigma _H$.

*Summary and discussion* In sum, we have a relatively simple model $\varSigma _H$ of cell-to-cell signaling, specifically, a model (23) from conductance to conductance with only three parameters $\rho$, $\mu$, and $\tau _{\zeta }$, that, through an appropriate choice of intermediate neuron model and its inverse, results in striking agreement with the measurements. Such simplified models of complete signal paths can be used in Hopfield-like network models with feedback connections and are likely to reduce the computational cost in network simulations. Furthermore, in the case of the squid, the high-level model provides us with a clear, but tentative, description of what the particular signal path does from a functional, input–output point of view: it realizes an escape threshold that enables the animal to evade predators. The given neuron model and the derived synapse model describe in more detail how this behavior is realized physically. The assumptions made are relatively mild and their number relatively small, not only for the field of neuroscience, but also for the obtained level of abstraction. Furthermore, in this particular example, these assumptions are for the most part supported by the results.

Of course, we cannot expect the derived model to fit detailed data sets exactly, and, although the qualitative agreement is striking, there are still some quantitative discrepancies left. For instance, the peak potential of the neuron model is still too high (approximately 190 mV) and the responses in Fig. 7 appear ‘wider’ or ‘broader’ than those in Fig. 8. The reason for these discrepancies can be traced back to either our threshold hypothesis, perhaps the most likely suspect, or to one of our two assumptions: (1) that a FitzHugh-type model suffices for a second-order giant and (2) that we can lump together all synaptic conductances. On the other hand, it may still be that we have not yet found the right parameters. What ever the case may be, other hypotheses can be drawn from the class (19) for verification, other neuron models can be drawn from the class (9), and distinct synaptic conductances are covered next.

#### Remark 4

One could also consider a signal path from potential to potential or from transmitter concentration to transmitter concentration. The reason for choosing a path from conductance to conductance is that, for excitable cells, we expect the synaptic conductance to be the ‘smoothest’ or most gradual signal representation in the chain and therefore the easiest to ‘read’ or interpret by humans. The ‘diffuse’ transmitter concentration may also be a good candidate, while, for graded neurons without impulses, a path from potential to potential may be more appropriate for interpretation.

#### Remark 5

Our reasoning for using three intermediate variables in (23) is as follows. Consider the synaptic data from Augustine et al. (1985), which is faithfully redrawn in Fig. 7 for our convenience. Note that although there are ‘jumps’ or ‘steps’ in the presynaptic depolarizations $\upsilon (t)$ of the membrane potential, there are no jumps or steps in the resulting postsynaptic response *y*(*t*), which we can take to be proportional to the postsynaptic current, cf. Eq. (29). Modeling the synapse with a direct function $y=\varphi (\upsilon )$ would cause the modeled synaptic response to have jumps or steps too. Modeling the synapse with a first-order system ${\dot{y}} = \varphi (y,\upsilon )$ would result in a response with sharp corners. Hence, the ‘smooth’ response of the synapse suggests using a system $\ddot{y}= \varphi (y,{\dot{y}}, \upsilon )$ of at least second order, or a state-space model of relative degree at least two (Sect. 4.3). This, then, is our modeling *choice*, which could be relaxed, if, e.g., one does not care about corners in the response. Note now that the full cascade includes a neuron model of relative degree one, that is, one has to differentiate the output $\upsilon$ of the neuron model once with respect to time in order for its input *u* to appear explicitly. Hence, since neuron and synapse are connected in series, the hypothetical high-level model should have a relative degree of at least three, and as one can see one indeed has to differentiate the output *y* of the model (23) three times in order for the input *u* to appear explicitly.

#### Remark 6

Since our method is mainly about model *construction*, quantitative criteria for model validation are beyond the scope of the present article. Hence, we tuned the model parameters by hand. Nevertheless, we do give some pointers here on how to use existing methods for parameter tuning and validation (van Geit et al. 2008). One approach would be as follows.

- Use existing methods to fit the given model to its data.
- Fix the parameters obtained in step (a). (Note that these, by construction, are shared by the complementary model).
- Use the *few* remaining free parameters, i.e., those of the simple, high-level model, to fit the complementary model to its data.

Note that, since we cannot expect (simplified) models to fit data sets exactly, and since data sets are usually obtained independently in different experimental setups, we may want some more freedom. To this end, we note again that the complementary model and the given model, by construction, are constrained to share some of their parameters. Another alternative approach would now be as follows. Temporarily remove this shared parameter constraint and use existing methods to tune both the given model and the complementary model *independently* first. Then, ‘move’ each of the parameters of the given model as far as possible in the direction of its corresponding value in the complementary model without loosing the essentials of its behavior, that is as far as the tolerance of the performance measure allows. (There are most likely several allowed parameter combinations that form a continuous set or manifold). Next, fix these parameters in both the given model and the complementary model, i.e., use them as new parameters for the complementary model as well. And finally, re-tune the few parameters of the high-level model to fit the adjusted complementary model to its data again, as in step (c) above.

### Distinct excitatory and inhibitory inputs

**Fig. 9:**
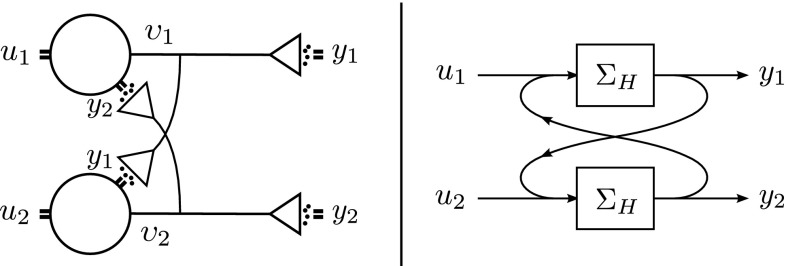
*Left* a schematic representation of a (minimal) conductance-to-conductance network with feedback connections. *Right* models of complete signal paths can be used as building blocks in the construction of network models with feedback connections. In the high-level network model, some details no longer appear, in this case the intermediate voltage responses and transmitter concentrations. Only the input–output essentials are retained

So far we have considered neuron models with only one synaptic reversal potential. However, even though their corresponding high-level models can already be used in networks with feedback connections (Fig. 9), we need to consider neuron models with at least two synaptic reversal potentials, in order to allow for simultaneous excitatory and inhibitory inputs. Our method indeed also allows for this, as we show in the appendix. There, we generalize the case where a model of the first subsystem is given, so as to include multi-input multi-output systems (Sect. A.1). We then consider a given model consisting of a pair of neurons with two distinct input conductances each and derive an interesting class of high-level models for which a complementary synaptic system can be derived explicitly (Sect. A.2). To keep the presentation of our method as transparent as possible, however, we do not include these natural and necessary extensions in the main body of the text; instead, we focus in the next section on the case where a model of the second subsystem is given.

## The second subsystem is given (from transmitter release to networks)

In this section, we cover the case where a model of the second subsystem is given. We start with a neuronal example, emphasizing further how our method can be used to circumvent unknown mechanisms of synaptic transmission. We then address the general multi-input multi-output case.

**Fig. 10:**
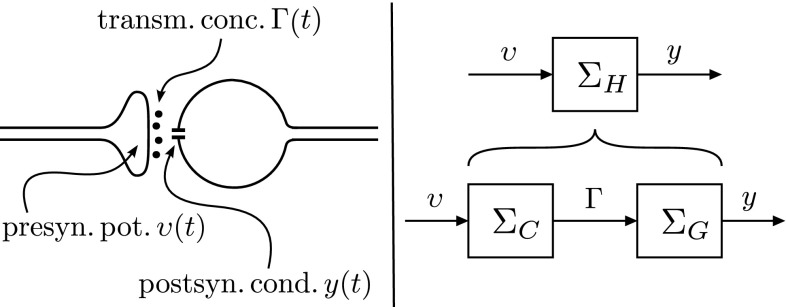
*Left* a simplified schematic representation of synaptic transmission. Fluctuations in presynaptic membrane potential $\upsilon (t)$ can bring about the release of neurotransmitter into the synaptic cleft, effecting the transmitter concentration $\varGamma (t)$ in the cleft. The transmitter may bind to receptors or receptor channels in the postsynaptic membrane. This can lead to the opening or closing of ion channels, which in turn will alter the membrane conductance *y*(*t*) of the postsynaptic neuron. *Right* synaptic transmission viewed as a cascade of subsystems: voltage-dependent release $\varSigma _C$ and transmitter-dependent conductance $\varSigma _G$

### Unknown mechanisms of transmitter release

As a first example, we use our method to deal with unknown mechanisms of transmitter release (Fig. 10). We assume that a suitable or satisfactory model of transmitter-dependent, postsynaptic conductance *y*(*t*) is given. Our aim is to establish a class of high-level models $\varSigma _H$ from presynaptic potential $\upsilon (t)$ to postsynaptic conductance *y*(*t*), for which we can explicitly derive a ‘physical’ state-space realization of the complementary model of transmitter release, that is, the model from presynaptic potential $\upsilon (t)$ to transmitter concentration $\varGamma (t)$. Note that in this case the high-level model does not *yet* represent a complete signal path from conductance to conductance, from membrane potential to membrane potential, or from transmitter concentration to transmitter concentration.

*The given model: transmitter-dependent postsynaptic conductance* Let the given model for transmitter-dependent, postsynaptic conductance *y*(*t*) be based on the reaction kinetics of a postsynaptic receptor channel, where we assume that the conductance *y*(*t*) is proportional to the fraction of channels that are in the open, conducting state. In a simple two-state model, *n* transmitter molecules *T* bind to the receptor channel according to the following scheme: 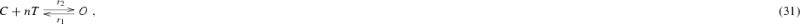


where *C* denotes the closed state of the channel, *O* denotes the open state and $r_1 , r_2 > 0$ denote reaction rates (Abbott 1994; Dayan and Abbott 2001; Destexhe et al. 1994). The associated evolution equation for the above scheme is given by: 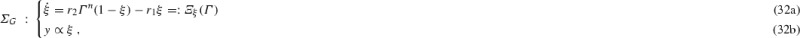


where the fraction $\xi (t)$ of receptor channels in the open state *O* takes values between zero and one, that is, $0<\xi (t)<1$, the fraction of channels in the closed state *C* is represented by $1-\xi$, and the transmitter concentration is represented by $\varGamma (t) \ge 0$.

Since we assume that the output conductance *y*(*t*) is proportional to $\xi (t)$, it is convenient to choose the unit of conductance such that $y=1 \cdot \xi$, so that the inverse of the given model $\varSigma _G$ above reads:

33 $$\begin{aligned} \varSigma ^{-1}_G : \varGamma = \varXi _{y}^{-1}({\dot{y}}) = \left\{ \frac{{\dot{y}} +r_1 y}{r_2(1-y)} \right\} ^{1/n} . \end{aligned}$$

This inverse is valid if $\frac{\partial \varXi _{y}}{\partial \varGamma } = n r_2 (1-\xi ) \varGamma ^{n-1} \ne 0$, that is, if either $n=1$ or else $\varGamma \ne 0$, or in biological terms if:

1. either the number of transmitter molecules *n* needed to open a channel is only 1, or else,
2. the transmitter concentration $\varGamma$ is never zero, i.e., there is always some residual transmitter left in the synaptic cleft.

*High-level models with explicit realizations* We will demonstrate that a ‘physical’ state-space realization of the complementary model $\varSigma _C$ of transmitter release can be derived explicitly, if we choose our hypothetical, high-level model $\varSigma _H$ of synaptic transmission from a general class of models. The proposed class consists of models of the form: 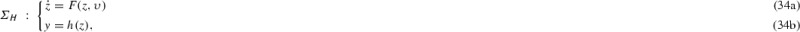


where *z*(*t*) is a suitably initialized state of arbitrary dimension, $F(\, . \, ,\upsilon )$ is an appropriate vector field parameterized by the presynaptic potential $\upsilon (t)$, and the map *h* is such that the postsynaptic output conductance takes values between zero and one, i.e., such that $0< y(t) < 1$. Recall that, even though the form (34) is very general, the aim is to keep the hypothesis $\varSigma _H$ as simple as possible.

*The complementary model of transmitter release* We can now derive a realization of the complementary model of transmitter release. Using (34), we can replace each *y* in (33) with *h*(*z*) and each ${\dot{y}}$ with $\frac{\partial h}{\partial z}F(z,\upsilon )$ to obtain a realization of the complementary model $\varSigma _C = \varSigma _G^{-1} \circ \varSigma _H$ of transmitter release. An explicit state-space realization is then given by: 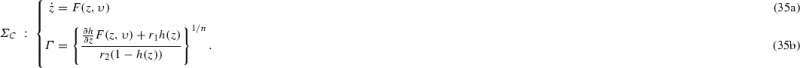


In sum, the nonlinear cascade $\varSigma _G \circ \varSigma _C$ reduces to $\varSigma _H$, and one is still free to postulate any hypothetical, high-level model $\varSigma _H$ of the form (34).

We could now, in principle, verify whether or not the derived model of transmitter release $\varSigma _C$ is consistent with some postulated, hypothetical model $\varSigma _H$ of synaptic transmission, assuming of course that the given model of transmitter-dependent conductance $\varSigma _G$ is satisfactory. To do so requires measurements. In particular, it requires the measured time courses of transmitter concentration $\varGamma (t)$ elicited by presynaptic potentials $\upsilon (t)$ in measurement pairs $(\upsilon (t),\varGamma (t))$. Although we will not verify a hypothesis here, it is interesting to note that, in the case of the squid giant synapse, the previously derived synapse model (28) can immediately be written in the biologically more detailed form: 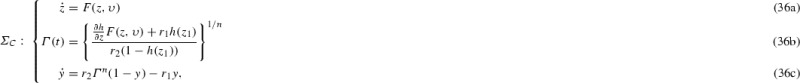


where the intermediate transmitter concentration $\varGamma (t)$ now appears explicitly, that is of course, if the given model based on the kinetic scheme (31) is indeed satisfactory for the squid giant synapse. This can be seen by noting that the model (28) is of the form (34). In other words, the cascade consisting of the biologically detailed complementary model (36) and the given neuron model (20) reduces to the simple, hypothetical model (23), and yet, it can still be related to the highly detailed signal path depicted in Fig. 1. Note that the full cascade now consists of *three* subsystems. The models for the first and the last subsystem (the neuron and the transmitter-dependent conductance) are, respectively, given beforehand by (20) and (36c), and we essentially derived the complementary model for the middle subsystem, that is, the model of transmitter release (36a, 36b) from presynaptic potential $\upsilon (t)$ to transmitter concentration $\varGamma (t)$.

Although this model of release itself remains abstract, this is acceptable given that many mechanisms of release are still questioned, debated or unknown (remark 1). Furthermore, the above suggests that our method can be used to fill in such holes in present knowledge step by step as more and more details become known.

### The general case (Fig. 3)

We now consider for completeness the more general, multi-input multi-output case. Consider a physical system that can be thought to consists of two subsystems connected in series. Again, our aim is to establish a simple, high-level model $\varSigma _H$, describing the behavior of the full system from a functional, input–output point of view. We assume that a satisfactory model $\varSigma _G$ of the second subsystem is already given, describing in more detail how part of the system is realized (Fig. 3). What is thus still required in order to complete the cascade is a complementary model $\varSigma _C$ of the first subsystem.

*The form of the given model and its inverse* Since we intend to use the inverse of the given model in our derivation, we make a few assumptions. We assume that the given model $\varSigma _G$ is such that (possibly after an appropriate state transformation) it is in the normal form (Nijmeijer and van der Schaft 1990): 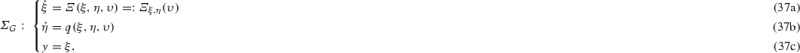


where the input $\upsilon (t)$, the output *y*(*t*), and the external state $\xi (t)$ are all *d*-dimensional variables, the internal state $\eta (t)$ is an *n*-dimensional variable, $\varXi$ is sufficiently smooth, and the square matrix $\frac{\partial \varXi _{\xi ,\eta }(\upsilon )}{\partial \upsilon }$ is nonsingular, in a neighborhood of the points of interest. The system is initialized in a suitable state. By the implicit function theorem, again assuming its conditions hold, an inverse system is then implicitly given by: 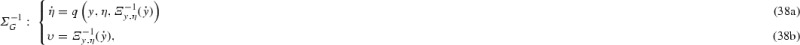


where now *y*(*t*) is the input, $\upsilon (t)$ is the output, and $\eta (t)$ is the suitably initialized state, cf. Nijmeijer and van der Schaft (1990). We assume again that the inverse system, driven by reasonable *y*(*t*) and ${\dot{y}}(t)$, is sufficiently stable, e.g., exponentially stable, so that solutions with different initial conditions all converge to the same solution, cf. Eqs. (10a) and (11). Systems with this property are said to be (exponentially) convergent, for the admissible class of inputs, cf. (Pavlov and Petterson 2008; Pavlov et al. 2004). Again, this convergence assumption may seem restrictive, but ‘slow’ recovery processes, such as tonic adaptation, seem ubiquitous in neurobiological systems. Note the input derivatives ${\dot{y}}$ in the inverse system above. We claim that this inverse system does not have a classical state-space realization.

*The general form of the high-level model* We will demonstrate that the complementary model $\varSigma _C$ is *guaranteed* to have a ‘physical’ state-space realization if we choose our hypothetical, high-level model $\varSigma _H$, meant to describe the full system’s functional behavior, from a general class of models. In fact, as we will see, when $\varSigma _G$ is of a slightly less general form, such realizations can be derived explicitly. The proposed class consists of models of the form: 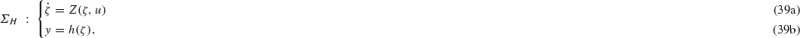


where $\zeta (t)$ is a suitably initialized, *m*-dimensional state, $Z(\, . \, ,u)$ is an appropriate, *m*-dimensional vector field parameterized by an input *u*(*t*) of arbitrary dimension, and *h* is a sufficiently smooth map, resulting in a *d*-dimensional output *y*(*t*). Recall that, even though the form (39) is very general, the aim is to keep the hypothesis $\varSigma _H$ as simple as possible.

*The complementary model* We can now derive a realization of the complementary model. Using (39), we can replace each *y* in (38) with $h(\zeta )$ and each ${\dot{y}}$ with $\frac{\partial h}{\partial \zeta }Z(\zeta ,u)$ to obtain a realization of the complementary model $\varSigma _C = \varSigma _G^{-1} \circ \varSigma _H$. The realization is implicitly given by: 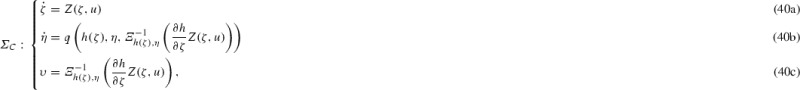


with state $z = (\zeta _1, \ldots , \zeta _m, \eta _1, \ldots , \eta _{n})$, input *u*(*t*), and output $\upsilon (t)$. This system is clearly of the form (2). Hence, with hypotheses $\varSigma _H$ of the form (39), our derived model $\varSigma _C$ is *guaranteed* to have a realization and therefore satisfies our ‘physical’ realizability constraint. Whether or not we can make this realization explicit, depends on the particular $\varXi$. For instance, when it is of the form $\varXi _{\xi ,\eta }(\upsilon )=\beta (\xi ,\eta ) + \alpha (\xi ,\eta ) \upsilon$, one can, in *principle*, derive $\varXi _{\xi ,\eta }^{-1}(.)$ explicitly, although it may still be computationally intensive for large systems.

In sum, the nonlinear cascade $\varSigma _G \circ \varSigma _C$ reduces to $\varSigma _H$, and one is still free to postulate any hypothetical, high-level model of the form (39) by specifying *Z* and *h*, where of course the aim is to keep $\varSigma _H$ as simple as possible. In the appendix (Sect. A.3) we explain how this applies to voltage-to-voltage networks where the given model of the second subsystem consists of several neurons with ion-specific input conductances.

### Final remarks on concepts from control theory

In this paper, we have made use of results in the literature on nonlinear control systems (see, e.g., Terrell (1999a, b) and Khalil (2002) for some of the fundamentals). For instance, both the form (37) above and the form (41) below are *normal forms* for systems with the same number of inputs as outputs, so-called *square systems* (with uniform relative degree one, to be more specific, as we explain shortly). In other words, many systems can be transformed into the forms (41) and (37) by an appropriate (local) coordinate transformation. The normal form (41) is limited to *control-affine* systems, i.e., systems that are linear, or better affine, with respect to the input, cf. Isidori (1995), while the form (37) is its direct generalization to systems that are not necessarily control-affine, cf. Nijmeijer and van der Schaft (1990). These normal forms are particularly convenient for *systems inversion*, and it is interesting to note that essentially all single-compartment, conductance-based neuron models with input current or conductance and output potential are already in normal form. This includes such models as the Hodgkin–Huxley model (Hodgkin and Huxley 1952) and its reductions (FitzHugh 1961; Rinzel 1985; Kepler et al. 1992).

We have made extensive use of inverse systems, and these play an important role in *feedback linearization* (Terrell 1999b; Isidori 1995), i.e., linearization of nonlinear input–output systems by means of a state feedback, where, with the aid of the above normal forms, the inverse system is used (often implicitly) to cancel out the nonlinearities. The stability of the inverse system is important in this context and is determined by the stability of the *internal dynamics* of the system under consideration. In control, when the desired output is a fixed set point, the internal stability can be checked locally with the aid of the so-called *zero dynamics* (Isidori 1995), and systems with stable zero dynamics are often termed (locally) *minimum phase*, cf., e.g., Khalil (2002). Our use of inverse systems, however, has more in common with their use in the problem of reproducing a reference output, where the reference output is a trajectory, and where the inverse is used to achieve exact (or asymptotic) tracking, cf. Isidori (1995). In this case, the internal stability of the system along the reference output trajectory must be taken into account. The corresponding internal dynamics are sometimes called the *tracking dynamics* (Pavlov and Petterson 2008), the *reference dynamics* (Zhao and Chen 1998), or the *forced* zero dynamics (Henson and Seborg 1997), and they coincide with the dynamics of the inverse system when driven by the reference output trajectory. In this paper, we *assumed* these to be sufficiently stable for all trajectories.

Inverse models can introduce input derivatives and are, in general, not *realizable*. Realizability conditions for systems with input derivatives can be found in Freedman and Willems (1978) and are generalized in Delaleau and Respondek (1995). A constructive procedure for finding a realization of a system with input derivatives (if it exists) can be found in Glad (1989). It involves *generalized* or *extended* state transformations, and to ensure realizability, when postulating high-level models, we must stress to keep an important invariant of state transformations in mind: the *relative degree*. For single-input single-output systems, the relative degree is the number of times *r* that one has to differentiate the output with respect to time in order for the input to appear explicitly. Similarly, for multi-input multi-output systems, each $r_i$ in the vector relative degree $\{r_1, \ldots , r_d\}$ of the system represents the number of times one has to differentiate the *i*th output in order for *one* of the inputs to appear explicitly. In our approach, the relative degree of the given model cannot exceed that of the high-level model. For instance, the general forms (39) and (43) are deliberately chosen such that both have a vector relative degree $\{r_1,\ldots ,r_d\}$ with $r_i \ge 1$. Our reasoning is as follows. Since the given model and the complementary model are connected in series, and since the given models (37) and (41) have uniform relative degree one, it follows that one has to differentiate each individual output of the full cascade *at least* once in order for one of the inputs to appear explicitly. The relative degree is also sometimes called the relative order (Tsinias and Kalouptsidis 1983) or the characteristic number (Nijmeijer and van der Schaft 1990). For more on the relative degree, see, e.g., Terrell (1999b); Isidori (1995); Tsinias and Kalouptsidis (1983); Henson and Seborg (1997); Nijmeijer and van der Schaft (1990).

## Conclusion

In this paper, we have introduced a novel simplification method for dealing with physical systems that can be thought to consist of two subsystems connected in series, such as a neuron and a synapse. The aim of our method is to facilitate finding a simple, yet convincing model of the full cascade-connected system, assuming that a satisfactory model of one of the subsystems is already known, or better given. Our method can be summarized as follows. First postulate a simple, hypothetical model of the full system and then use the inverse of the given model to derive a model of the remaining, unidentified subsystem. For instance, given a neuron model, derive a synapse model based on a simple, hypothetical model of cell-to-cell signaling. The derived model can then be verified against measurements to either support or reject the hypothesis. The necessary tools are provided by nonlinear systems theory. The results from our squid example suggest that simple, yet faithful models of cell-to-cell signaling are feasible with our method.

There are several advantages to our approach. These stem from the fact that our method exploits the relationship between the full system and its subsystems. For instance, our method promotes simplicity. By keeping the hypothetical, high-level model as simple as possible, i.e., by keeping the number of variables and parameters to a minimum and by trying models with a simple interpretation first, our method aims to summarize data with theory. If successful, the achieved reduction not only provides us with insight, it will also, most likely, facilitate further analysis. In theoretical neuroscience, this is particularly useful since the analysis of networks of modeled neurons can quickly become prohibitively complex. In addition, another reason for favoring simple models is to avoid overfitting. Indeed, due to the fact that the complementary model is derived from a simple, high-level hypothesis, it will, in general, be heavily constrained, which is exactly what we want. In fact, the complementary model is not only constrained by its *own* data, but also by the hypothetical model, the given model and *its* data. Finally, unlike the more obvious component-wise approach (Sect. 1.3), our approach is *not* prone to misinterpretation. The models of the subsystems are no longer obtained independently. In fact, they form a matching pair. Small modeling errors or slightly suboptimal parameters are no longer amplified by nonlinearities, but instead, the complementary model compensates for small discrepancies in the given model by utilizing the inverse of the given model.

## Figures and Tables

**Fig. 11:**
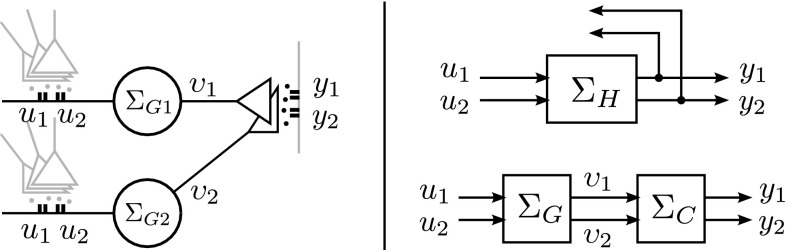
*Left* a schematic representation of a minimal canonical circuit with input conductances *u*(*t*) and output conductances *y*(*t*). Each neuron ‘receives’ two distinct input conductances $u_1$ and $u_2$ from other neurons or receptors, and their distinct output conductances $y_1$ and $y_2$ contribute to the total specific input conductances of other cells. *Right* the circuit represented as a cascade of subsystems together with its high-level representation. Such high-level models can be used as building blocks in the construction of networks with feedback connections

**Fig. 12:**
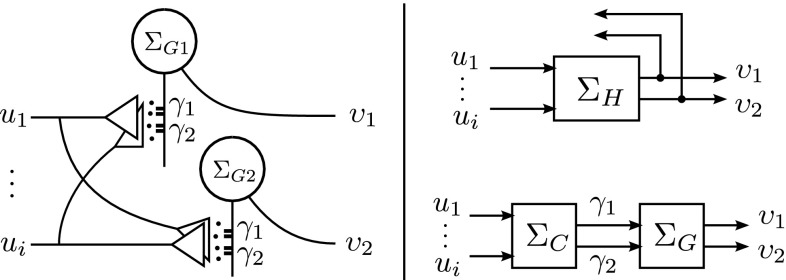
*Left* a schematic representation of a minimal canonical circuit with input potentials *u*(*t*) and output potentials $\upsilon (t)$. Each neuron ‘receives’ two distinct input conductances $\gamma _1$ and $\gamma _2$ from an arbitrary number of neurons. *Right* the circuit represented as a cascade of subsystems together with its high-level representation. Such high-level models can be used as building blocks in the construction of networks with feedback connections

## A Appendix

In this appendix, we first extend the case where a model of the first subsystem is given (Fig. 2) to the more general, multi-input multi-output case. This allows us to study neuron models with both excitatory and inhibitory inputs, and hence, as an example, we consider a given model consisting of a pair of neurons with two distinct input conductances each. For this given model, we derive a class of high-level models for which a complementary synaptic system can be derived explicitly and from which hypotheses can thus be drawn for verification. Next we similarly apply the general result obtained in Sect. 4.2 to voltage-to-voltage networks where the given model of the second subsystem consists of several neurons with ion-specific input conductances.

### A.1 The first subsystem is given (general case)

Consider a physical system that can be thought to consist of two subsystems connected in series. Again, our aim is to establish a simple, high-level model $\varSigma _H$, describing the behavior of the full system from a functional, input–output point of view. We assume that a satisfactory model $\varSigma _G$ of the first subsystem is already given, describing how part of the system is realized (Fig. 2). What is still required is a complementary model $\varSigma _C$ of the second subsystem.

*The form of the given model and its inverse* As before, we will use the inverse of the given model in our derivation. Hence, we make a few assumptions. We assume that the given model $\varSigma _G$ is such that (possibly after an appropriate state transformation) it is in the normal form (Isidori 1995):

where the input *u*(*t*), the output $\upsilon (t)$, and the external state variable $\xi (t)$ are all *d*-dimensional variables, the internal state variable $\eta (t)$ is an *n*-dimensional variable, and the square matrix $\alpha$ is nonsingular, at the points of interest. The system is initialized in a suitable state $(\xi (0),\eta (0))$. An inverse system is then given by:

and where now *u*(*t*) is the output and $\upsilon (t)$ is the input, cf. Isidori (1995) (It is possible to eliminate the need for (42a) by replacing every occurrence of $\xi$ with $\upsilon$. However, when considering realizability later on, the present form is more convenient). We assume again that the forced system (42b), driven by reasonable $\xi (t)=\upsilon (t)$ and ${\dot{\upsilon }}(t)$, is sufficiently stable, so that solutions with different initial conditions all converge to the same solution, cf. Eqs. (10a), (11), and (38). Note the input derivatives ${\dot{\upsilon }}$ in the inverse system above. We claim that this inverse system does not have a classical state-space realization.

*The form of the high-level model* We will demonstrate that the realizability conditions for the complementary model $\varSigma _C$ are known, provided that we choose our hypothetical, high-level model $\varSigma _H$, meant to describe the full system’s functional behavior, from a particular class of systems. In fact, in the single-input single-output case, the resulting complementary model $\varSigma _C$ is *guaranteed* to have a realization. The class consists of systems of the form:

where $\zeta (t)$ is the suitably initialized, *m*-dimensional state, *u*(*t*) is the *d*-dimensional input, and the output *y*(*t*) has arbitrary dimension. The map *f* and the columns $g_i$ of *g* are appropriate, *m*-dimensional vector fields, and *h* is the readout map. Recall that, although the form (43) is very general, the aim is to keep the hypothesis as simple as possible.

*The complementary model* Combining Eqs. (42) and (43), we find that the complementary model $\varSigma _C = \varSigma _H \circ \varSigma _G^{-1}$ is given by:

with suitable initial conditions $(\zeta (0),\eta (0),\xi (0))$.

Unfortunately, this system is not of the form (1); it still contains input derivatives ${\dot{\upsilon }}$. Hence, it is not immediately obvious whether or not this system has a state-space realization and satisfies our ‘physical’ constraint.

*Realizability of the complementary model* Results on the realizability of systems of the form (44) have already been reported in the literature. With $\varXi _{\xi ,\eta }^{-1} ({\dot{\upsilon }})$ as in (42d), the model is of the form:

45 $$\begin{aligned} \varSigma _C : \; {\dot{x}} = a(x) + b(x) {\dot{\upsilon }} \, , \quad y=h(x_1, \ldots , x_m) \end{aligned}$$

where *x*, *a*(*x*), and *b*(*x*) read as follows:

46 $$\begin{aligned} x= & {} \left[ \begin{array}{c} \zeta \\ \eta \\ \xi \end{array} \right] \in \mathbb {R}^m \times \mathbb {R}^{n} \times \mathbb {R}^d \; , \end{aligned}$$

47 $$\begin{aligned} a(x)= & {} \left[ \begin{array}{c} f(\zeta ) - g(\zeta )[\alpha (\xi ,\eta )]^{-1}\beta (\xi ,\eta ) \\ q(\xi ,\eta ) -p(\xi ,\eta )[\alpha (\xi ,\eta )]^{-1}\beta (\xi ,\eta ) \\ 0 \\ [-1ex] \vdots \\ 0 \end{array} \right] \; , \end{aligned}$$

and

48 $$\begin{aligned} b(x)=\left[ \begin{array}{c} g(\zeta )[\alpha (\xi ,\eta )]^{-1} \\ p(\xi ,\eta )[\alpha (\xi ,\eta )]^{-1} \\ \begin{array}{ccc} 1 &{} \cdots &{} 0 \\ [-1ex] \vdots &{} \ddots &{} \vdots \\ 0 &{} \cdots &{} 1 \end{array} \end{array} \right] \; . \end{aligned}$$

The realizability conditions for (45) are given in Freedman and Willems (1978), and in the single-input single-output case ($d=1$) when *b* consists of a single column, the complementary model is *guaranteed* to have a classical state-space realization of the form (1) and hence satisfies our ‘physical’ constraint. When *b*(*x*) consists of two columns $b_1(x)$ and $b_2(x)$, for instance, then these are required to satisfy the following realizability condition:

49 $$\begin{aligned} \frac{\partial b_2}{\partial x} b_1 = \frac{\partial b_1}{\partial x} b_2 \; . \end{aligned}$$

*Explicit realizations through state transformations* Even if a classical state-space realization (1) is guaranteed to exist, it is generally hard to actually find such a realization given (44) or (45). Fortunately, for verifications of hypotheses, explicit realizations are not required, numerical verifications suffice. One may, however, attempt to find a realization by seeking a smooth coordinate transformation:

50 $$\begin{aligned} \left[ \begin{array}{c} z(x) \\ \xi \end{array} \right] = \left[ \begin{array}{c} \varPsi _{\xi }(\zeta ,\eta ) \\ \xi \end{array} \right] \; , \end{aligned}$$

so that in the new evolution equations for $z \in \mathbb {R}^{m+n}$, given by:

51 $$\begin{aligned} {\dot{z}} = \frac{\partial z}{\partial x} \left[ a(x) + b(x) {\dot{\upsilon }}\right] , \end{aligned}$$

the ${\dot{\upsilon }}$-terms (or ${\dot{\xi }}$-terms) cancel. That is, one attempts to find a solution *z*(*x*) to the system of PDE’s:

52 $$\begin{aligned} \frac{\partial z}{\partial x} b(x) = \mathbf {0}_{k \times d} \; , \end{aligned}$$

where $k=m+n$ and where $z(x)=\varPsi _{\xi }(\zeta , \eta )$ is such that the mapping (50) is invertible:

53 $$\begin{aligned} x = \left[ \begin{array}{c} \zeta \\ \eta \\ \xi \end{array} \right] = \left[ \begin{array}{c} \varPsi _{\xi }^{-1} (z) \\ \xi \end{array} \right] . \end{aligned}$$

One is left with a state-space realization:

of the form (1), where the replacement $\xi =\upsilon$ has been made and the equation ${\dot{\xi }} = {\dot{\upsilon }}$ in (44) has now become redundant. The coordinate transformation (50) is an example of an *extended* state coordinate transformation, cf. Kotta and Mullari (2005) for the single-input single-output case and Kotta and Mullari (2006) for the multi-input multi-output case.

*Numerical verification* As already mentioned, for verifications of hypotheses, we may have to resort to numerical verification. Although, in this paper we derive realizations explicitly, in the case where a model of the first subsystem is given, such realizations are, as already mentioned, generally hard to find, due to the fact that they involve solving a system of PDE’s. Fortunately, once the *existence* of a realization has been established, numerical verifications suffice. For instance, the discrete time analog of (45):

55 $$\begin{aligned} \frac{x_{t+\tau }-x_t}{\tau } = a(x_t) + b(x_t) \frac{\upsilon _{t+\tau } - \upsilon _t}{\tau } \; , \end{aligned}$$

where $\tau$ is the time step, and $x_t=(\zeta _t,\eta _t,\xi _t)$, is consistent with the use of the forward Euler method for the given model $\varSigma _G$:

56a $$\begin{aligned} \xi _{t+\tau }&= \xi _t + \tau \left\{ \beta (\xi _t, \eta _t) + \alpha (\xi _t, \eta _t) u_t \right\} \end{aligned}$$

56b $$\begin{aligned} \eta _{t+\tau }&= \eta _t + \tau \left\{ q(\xi _t, \eta _t) +p(\xi _t,\eta _t) u_t \right\} . \end{aligned}$$

Note that (55) depends on future values $\upsilon _{t+\tau }$ of the input. If so desired, it can be given a *causal* numerical realization:

57a $$\begin{aligned} x_{t+\tau }&= \widetilde{F}(x_t,\upsilon _t) = x_t + \tau a(x_t)+b(x_t)(\upsilon _t-\xi _t) \end{aligned}$$

57b $$\begin{aligned} y_t&= \widetilde{H}(x_t,v_t) = h(\zeta _{t+\tau }) . \end{aligned}$$

Note that the state update (57a) is now delayed by one time step ($\xi _t=\upsilon _{t-\tau }$) compared to (55) and that the output equation (57b) compensates for this delay with:

58 $$\begin{aligned} \zeta _{t+\tau } = \left( \widetilde{F}_1(x_t,\upsilon _t), \ldots , \widetilde{F}_m(x_t,\upsilon _t) \right) . \end{aligned}$$

This numerical method was used for both Figs. 6 and 8.

### A.2 Conductance-to-conductance networks

As an example of the above general case, we now consider conductance-to-conductance networks where the given model of the first subsystem consists of several neurons with ion-specific input conductances.

*A given model consisting of several neurons* In order to extend our result for neuron models of the form (9), so that it includes neuron models that allow for distinct, ion-specific input conductances, we need to increase the number of outputs. For left invertibility, the given model needs to have at least as many outputs as inputs. This results in a given model of the form:

where the index *i* runs over the number of neurons *d* and the index *j* runs over the number $N \le d$ of distinct, synaptic reversal potentials per neuron. Each synaptic conductance $u_j \ge 0$ with dimensionless efficacy or strength $w_{ij} \ge 0$ represents the total, summed conductance, with specific driving force $p_{ij} -\upsilon _i$, received from other neurons or receptors, cf. (9). Here, we take the number of inputs $u_j$ and the number of outputs $\upsilon _i$ to be the same for convenience, i.e., $N=d$. In vector form, the model reads:

where the output $\upsilon (t)$ and the input *u*(*t*) are both *d*-dimensional, and $\eta (t)$ is $(n \cdot d)$-dimensional, with *n* the collective number of activation and inactivation variables per neuron. Its inverse is given by:

where now $\upsilon (t)$ is the input and *u*(*t*) is the output, provided of course that $W(\upsilon )$ is nonsingular. We assume the $\eta$-dynamics to be sufficiently stable, cf. Eq. (11). The problem we want to address is as follows. What class of hypothetical high-level models $\varSigma _H$, from input conductances *u* to output conductances *y*, would result in a realizable complementary model $\varSigma _C$?

*The high-level model* The above problem may be too ambitious to solve in general. However, the least we can do is establish a class of hypothetical high-level models that could be realized, in *principle*, by a pair of neurons with two distinct input conductances each (Fig. 11). Such high-level models of canonical or local circuits can be used as building blocks for larger networks, in a way similar to Fig. 9. Hence, we restrict ourselves to the case where *W* is given by:

62 $$\begin{aligned} W(\upsilon ) = \left[ \begin{array}{ll} w_1(p_1-\upsilon _1) &{}\quad w_2(p_2-\upsilon _1) \\ w_1(p_3-\upsilon _2) &{}\quad w_2(p_4-\upsilon _2) \end{array} \right] , \end{aligned}$$

where $$w_1,w_2>0$$, and $$p_1, p_2, p_3$$ and $$ p_4$$ are such that $$W(\upsilon )$$ is nonsingular for the normal range of membrane potentials. Note that this allows for both excitatory and inhibitory inputs. Also note that, in this case, the complementary model represents a synaptic system that consists of at least two synapses, and, depending on the number of receiving neurons and synaptic sites, it may consist of many more. We first consider a subclass of high-level models consisting of models of the form:

where $\zeta (t)$ is a one-dimensional state, the maps $h_1$ and $h_2$ are such that the output conductances $y_1$ and $y_2$ are nonnegative, i.e., such that $y_i \ge 0$, and the specific input conductances $u_1$ and $u_2$ each represent a total input conductance resulting from other neurons or receptors. This time, parameters from the given model, namely the dimensionless strengths or efficacies $w_1$ and $w_2$, appear explicitly as parameters in the hypothesis (63), i.e., in terms of (43), we have $g(\zeta ) \equiv [w_1 \; w_2]$.

*The complementary model of the synaptic system* Combining Eqs.  (61) and (63), we find that the complementary model $\varSigma _C=\varSigma _H \circ \varSigma _G^{-1}$ is given by:

64a $$\begin{aligned} \overbrace{ {\dot{\zeta }} - C g \left[ W(\upsilon ) \right] ^{-1} {\dot{\upsilon }}}^{\displaystyle {\dot{z}}_k}&= f(\zeta ) + g \left[ W(\upsilon ) \right] ^{-1} I(\upsilon ,\eta ) \end{aligned}$$

64b $$\begin{aligned} {\dot{\eta }}&= q(\upsilon ,\eta ) \end{aligned}$$

64c $$\begin{aligned} y&=h(\zeta ) \; , \end{aligned}$$

with:

65 $$\begin{aligned} g[W(\upsilon )]^{-1} = \frac{w_1 w_2}{\det (W(\upsilon ))} \left[ \begin{array}{cc} p_4 \! - \! p_3&\quad p_1 \! - \! p_2 \end{array} \right] \; , \end{aligned}$$

for *W* as in (62), and with $\upsilon (t)$ the input potentials, and *y*(*t*) the output conductances (Fig. 11). As indicated by the ${\dot{z}}_k$-label above, this model can be given a state-space realization by changing coordinates.

*An explicit realization* In order to find a state-space realization for the above complementary model, consider the transformation $z=\varPsi _{\upsilon }(\zeta ,\eta )=(\eta ,\psi _{\upsilon }(\zeta ))$ where the last coordinate, the *k*th coordinate, is given by:

66 $$\begin{aligned} z_k=\psi _{\upsilon }(\zeta ) = \zeta + C \ln | \det (W(\upsilon )) | \end{aligned}$$

and the other *n* times *d* coordinates are given by $z_j=\eta _j \,$, hence, $k=n \cdot d + 1$. The inverse transformation $(\zeta ,\eta )=\varPsi ^{-1}_{\upsilon }(z)$ is specified by:

67 $$\begin{aligned} \zeta = \psi ^{-1}_{\upsilon } (z_k) = z_k - C\ln |\det (W(\upsilon ))| \; . \end{aligned}$$

This transformation (remark  7) results in an explicit realization given by:

or

69a $$\begin{aligned} {\dot{z}}&= \! \! \left[ \begin{array}{c} q(\upsilon ,z_1,\ldots ,z_{n d}) \\ \! f\left( \psi _{\upsilon }^{-1}(z_k)\right) + g \left[ W(\upsilon ) \right] ^{-1} \! \! I(\upsilon ,z_1,\ldots ,z_{n d}) \! \end{array} \right] \end{aligned}$$

69b $$\begin{aligned} y&= h \left( \psi _{\upsilon }^{-1}(z_k) \right) , \end{aligned}$$

which is clearly of the form (1). Note that, in general, the two distinct postsynaptic conductances $y_1$ and $y_2$ result from the presynaptic potentials $\upsilon _1$ and $\upsilon _2$ of both neurons. In sum, the nonlinear cascade $\varSigma _C \circ \varSigma _G$ reduces to $\varSigma _H$, and one is still free to postulate any hypothetical, high-level model of the form (63) by specifying *f*, $h_1$, and $h_2$.

It is straight forward to generalize the above results for the subclass of models of the form (63) to a more general class of high-level models of the form:

where $\zeta (t)$ is now an *m*-dimensional state and the output *y*(*t*) consists of an arbitrary number of distinct, specific output conductances. Recall however that the key is to keep the hypothetical, high-level model as simple as possible.

*Notes on verification* One of the main strengths of our method is that it can be used even when information is only partially available. Hence, instead of adding to the large body of data in the literature, we can use it to summarize such data with theory. Eventually however, we want to verify the unmeasured responses predicted by the hypothesis against data. If it is at all possible to find a circuit in neurobiology that corresponds well to our pair of neurons (Fig. 11) and that is suitable for experimentation, then, in order to verify the complementary model against synaptic data, there is still the problem of distinguishing between synaptic conductances with distinct reversal potentials. One approach could be to keep the presynaptic potential of one of the neurons at rest, and use pharmacological blockers to distinguish between distinct conductances in response to presynaptic depolarizations or hyperpolarizations of the other neuron.

#### Remark 7

Note that $\varPsi _{\upsilon }$ is a transformation in only a single coordinate (up to a reordering for convenient indexing) and that $z_k$ is a particular solution of the system of PDE’s (52), where *b*, corresponding to the expression (48) in (45), reads:

71 $$\begin{aligned} b(x)= \left[ \begin{array}{c} Cg[W(\upsilon )]^{-1} \\ \begin{array}{ll} 0 &{} 0 \\ [-1ex] \,\vdots &{}\,\vdots \\ 0 &{} 0 \\ 1 &{} 0 \\ [-0.5ex] 0 &{} 1 \end{array} \end{array} \right] ,\quad \text {with } x=(\zeta ,\eta ,\upsilon ) \; . \end{aligned}$$

Since $\partial b/\partial \eta \equiv 0$ (the derivative of the *i*th column of b with respect to $\eta$ satisfies $\partial b_i/\partial \eta \equiv 0$), this system of PDE’s is trivially satisfied by the other coordinates $z_j=\eta _j$, and the system reduces to:

72 $$\begin{aligned} \left[ \begin{array}{llllll} \frac{\partial z_k}{\partial \zeta }&\frac{\partial z_k}{\partial \eta _1}&\cdots&\frac{\partial z_k}{\partial \eta _{n d}}&\frac{\partial z_k}{\partial \upsilon _1}&\frac{\partial z_k}{\partial \upsilon _2} \end{array} \right] b(x) = \left[ \begin{array}{ll} 0&0 \end{array} \right] \; . \end{aligned}$$

Of course, one can check that the columns $b_1(x)$ and $b_2(x)$ of *b*(*x*) indeed satisfy the realizability condition (49).

### A.3 Networks from potential to potential

As an application of the general result in Sect. 4.2, we now consider voltage-to-voltage networks where the given model of the second subsystem consists of several neurons with ion-specific input conductances.

*A given model consisting of several neurons* For right invertibility, the given model needs to have at least as many inputs as outputs. Hence, we extend the model (9) to accommodate for this. This results in a given model of the form:

where the index *i* runs over the number of neurons *d* and the index *j* runs over the number *N* of distinct, synaptic reversal potentials per neuron. Each synaptic conductance $\gamma _j \ge 0$ with dimensionless efficacy or strength $w_{ij} \ge 0$ represents the total, summed conductance, with specific driving force $p_{ij} -\upsilon _i$, resulting from other neurons, cf. (9). Finally, for right invertibility we require $N \ge d$. (The latter may seem restrictive, but it is only required to hold for the high-level building block itself. By construction, our high-level models allow for feedback connections; hence, they can be wired-up into networks consisting of a large number of neurons). Here, we take the number of input conductances $\gamma _j$ and the number of output potentials $\upsilon _i$ to be the same for convenience, i.e., $N=d$. In vector form, the model reads:

where the output $\upsilon (t)$ and the input $\gamma (t)$ are both *d*-dimensional, and $\eta (t)$ is $(n \cdot d)$-dimensional, with *n* the collective number of activation and inactivation variables per neuron. Its inverse is given by:

where now $\upsilon (t)$ is the input and $\gamma (t)$ is the output, provided of course that $W(\upsilon )$ is nonsingular for the normal range of membrane potentials. The $\eta$-dynamics are assumed to be sufficiently stable, cf. Eq. (11). The problem we want to address is as follows. What class of hypothetical high-level models $\varSigma _H$, from input potentials *u*(*t*) to output potentials $\upsilon (t)$, would result in a realizable complementary model $\varSigma _C$?

*The general form of the high-level model* For a given model consisting of *d* neurons with *d* distinct input conductances each, we now establish a class of hypothetical, high-level models for which a state-space realization of the complementary model can be derived explicitly. A schematic representation is depicted in Fig. 12 for $d=2$, and for an arbitrary number of input potentials $u_i$. Note that this allows for neurons with both excitatory and inhibitory inputs. Also note that, in this case, the complementary model represents a synaptic system that consists of at least *d* synapses, and, depending on the number of input potentials $u_i$, it may consist of many more. Such high-level models of canonical or local circuits can be used as building blocks for larger networks. For instance, note that in Fig. 12 one can easily connect $\upsilon _1$ to $u_1$ so that $u_1=\upsilon _1$. Furthermore, for $d>2$ the number of possible canonical or local circuits quickly increases. The proposed class of realizable high-level models is quite general and consists of models of the form:

where $\zeta (t)$ is a suitably initialized state of arbitrary dimension, *u*(*t*) is an input of arbitrary dimension, and $\upsilon (t)$ is a *d*-dimensional output, cf. (39). The latter two both consist of membrane potentials. Recall that, even though $\varSigma _H$ is very general, the aim is to keep the hypothesis $\varSigma _H$ as simple as possible.

*The complementary model* We can now explicitly derive a realization of the complementary model. Using (76), we can replace each $\upsilon$ in (75) with $h(\zeta )$ and each ${\dot{\upsilon }}$ with $\frac{\partial h}{\partial \zeta }Z(\zeta ,u)$ to obtain a realization of the complementary model $\varSigma _C = \varSigma _G^{-1} \circ \varSigma _H$, which is explicitly given by:

77a $$\begin{aligned} {\dot{\zeta }}&= Z(\zeta ,u) \end{aligned}$$

77b $$\begin{aligned} {\dot{\eta }}&= q\left( h(\zeta ),\eta \right) \end{aligned}$$

77c $$\begin{aligned} \gamma&= \left[ W\left( h(\zeta )\right) \right] ^{-1} \! \left\{ C \frac{\partial h}{\partial \zeta } Z(\zeta , u) + I\left( h(\zeta ),\eta \right) \right\} , \end{aligned}$$

with *u*(*t*) the input potentials, $\gamma (t)$ the output conductances, and $(\zeta (t),\eta (t))$ the state. In sum, the nonlinear cascade $\varSigma _G \circ \varSigma _C$ reduces to $\varSigma _H$, and one is still free to postulate any hypothetical high-level model of the form (76).

*Notes on verification* One of the main strengths of our method is that it can be used even when information is only partially available. Hence, instead of adding to the large body of data in the literature, we can use it to summarize such data with theory. However, we do not only want to fit the available data; eventually, we also want to verify against measurements those responses of the complementary model that are *predicted* by the high-level hypothetical model. Although the explicit realization (77) allows for a wide range of hypotheses of the form (76), when it comes to verification, there is still the problem of distinguishing between synaptic conductances with distinct reversal potentials (if it is at all possible to find a circuit in neurobiology suitable for experimentation). Such distinctions are not always easily made experimentally. It is probably a good idea to start with a single graded, non-impulsive neuron (cf. remark 4) that is thought to be the target of two distinct neurotransmitters, and for which a satisfactory model with two distinct synaptic conductances is given. In other words, $d=1$, $N=2$ and $W(\upsilon )$ is of the form:

78 $$\begin{aligned} W(\upsilon ) = [ w_1(\upsilon ) \quad w_2(\upsilon )]. \end{aligned}$$

Hence, its inverse in (75b) represents a (non-unique) right inverse such as:

79 $$\begin{aligned} \left[ W(\upsilon )\right] _R^{-1}=\frac{1}{\Vert W(\upsilon )\Vert ^2} \left[ \begin{array}{c} w_1(\upsilon ) \\ w_2(\upsilon ) \end{array} \right] \; , \end{aligned}$$

so that $W(\upsilon )\left[ W(\upsilon )\right] ^{-1}_R=1$, for $\Vert W(\upsilon )\Vert ^2 \ne 0$. In this case, one approach could be to keep the postsynaptic potential $\upsilon$ of the neuron at rest, and use pharmacological blockers to distinguish between distinct conductances $\gamma _1(t)$ and $\gamma _2(t)$ in response to presynaptic depolarizations or hyperpolarizations *u*(*t*).
